# Prenylation of Flavanones by an Aromatic Prenyltransferase from *Fusarium globosum*

**DOI:** 10.3390/molecules30071558

**Published:** 2025-03-31

**Authors:** Dingtao Tang, Jiajie Quan, Zhengjiao Gao, Bingfeng He, Yu Hou, Peipei Fan, Meidong Pan, Jiali Yang

**Affiliations:** School of Food Science and Engineering, Hainan University, Haikou 570228, China; 22210832000018@hainanu.edu.cn (D.T.); 17637628953@163.com (J.Q.); 22220951350092@hainanu.edu.cn (Z.G.); 18107866739@163.com (B.H.); houyu0209@163.com (Y.H.); f15196524227@163.com (P.F.); 20223002169@hainanu.edu.cn (M.P.)

**Keywords:** enzyme catalysis, flavanone, substrate specificity, site-directed mutagenesis

## Abstract

Prenylation increases the structural diversity and biological activity of flavonoids. In this study, an aromatic prenyltransferase, FgPT1, was identified from *Fusarium globosum*. This enzyme was demonstrated to specifically catalyze the prenylation of flavanones, including naringenin, hesperitin, eriodictyol, liquiritigenin, rac-pinocembrin, and dihydrogenistein, and exhibited no activity toward other types of flavonoids, including chalcones, flavonols, isoflavonoids, and flavonols. Ultra-performance liquid chromatography-tandem mass spectrometry (UPLC-MS/MS) and nuclear magnetic resonance (NMR) analysis indicated that the majority of prenylated products were 6-*C* prenyl flavanones, with the exception of liquiritigenin, which was additionally transformed to 4′-*O* prenyl liquiritigenin. Enzyme kinetic analysis suggested that FgPT1 exhibited the highest catalytic efficiency towards naringenin, with a *k_cat_*/*K_M_* value determined as 61.92 s^−1^ M^−1^, and the lowest catalytic efficiency towards liquiritigenin, with a *k_cat_*/*K_M_* of 1.18 s^−1^ M^−1^. Biochemical characterization suggested that FgPT1 functioned as a metal-dependent enzyme with optimal activity in the presence of Ba^2+^ at pH 7.5 and 30 °C. Site-directed mutagenesis resulted in a series of mutants, including A325V with impaired prenylation activity and V116I, V181I, and V194I with enhanced activity. V194I displayed the highest enzymatic activity with a nine-fold increase compared to wild-type FgPT1.

## 1. Introduction

Flavanones are valuable bioactive compounds widely distributed in various plant species [1,2]. Prenyl flavanones represent a significant subclass, distinguished by the attachment of prenyl groups of varying chain lengths (C5, C10, C15) to the flavanone skeleton. This structural modification significantly improves membrane permeability and target binding capacity via increased lipophilicity [3,4]. Pharmacological studies have established the therapeutic potential of prenylated flavanones: Denticulain A exhibits selective cytotoxicity against colorectal cancer cells (IC_50_ = 46.08 μM for SW620; 56.83 μM for HCT-116) [5]. Notably, the phytoestrogen 8-prenylnaringenin effectively suppresses the proliferation of MCF-7 breast cancer cells and induces programmed apoptosis by selectively targeting and inhibiting the estrogen receptor-alpha (ER*α*) signaling pathway [6]. In terms of antioxidant activity, isoxanthohumol demonstrates significant DPPH radical scavenging capacity (EC_50_ = 8.38 mM), while the triply prenylated derivative ormosino exhibits more potent antioxidant activity (DPPH radical scavenging IC_50_ = 28.5 μM) [7,8]. Furthermore, Sekiguchi et al. reported that 6-prenylnaringenin alleviates neuropathic and visceral pain through T-type calcium channel blockade without inducing significant adverse effects [9]. These prenyl flavanones have been isolated from various plant tissues, including the flowers, fruits, and roots of *Fordia cauliflora*, *Humulus lupulus*, *Eysenhardtia texana*, *Erythrina variegata*, and *Dorstenia mannii*.

Despite the therapeutic promise of prenylated flavanones, their limited natural abundance presents significant challenges for pharmaceutical development. For instance, the dried cones of *Humulus lupulus* (hops) contain merely 0.1% (*w*/*w*) 8-prenylnaringenin, rendering natural extraction insufficient to meet the escalating market demand [10]. Moreover, conventional extraction processes are not only time-consuming and cost-prohibitive but also raise sustainability concerns due to excessive solvent consumption during isolation and purification steps, which contributes to ecological burdens and resource inefficiency [11]. Biosynthesis emerges as an eco-efficient strategy, capitalizing on enzyme-mediated regiospecificity under ambient conditions [12]. A critical aspect of biosynthesizing prenyl flavanones is the identification of aromatic prenyltransferases (PTs) that can utilize flavanones as substrates. Before that, plant-derived PTs like SfN8DT-1 (*C*8-specific) [13] and SfFPT (recognition of multiple substrates) [14] from *S. flavescens* demonstrated catalytic versatility. The LaPT2 derived from *Lupinus albus* also has substrate promiscuity, but it can only inefficiently catalyze the formation of unknown products from naringenin [15]. A fundamental constraint of plant-derived aromatic PTs is their membrane-bound nature, which renders them insoluble and poses challenges for heterologous expression—key limitations for biotechnological applications [16,17]. In contrast, microbial aromatic PTs (e.g., from bacteria and fungi) demonstrated superior solubility, high-yield production in *Escherichia coli*, and substrate flexibility toward flavonoids and other non-native compounds, making them a promising enzymatic tool for the synthesis of prenyl flavanones [18]. Notably, recent advances in microbial chassis engineering have demonstrated the feasibility of this approach. For instance, the integration of aromatic PTs (FPT and N8DT) into *Yarrowia lipolytica* enabled the construction of a yeast cell factory that successfully achieved a titer of 4.36 mg/L for 8-prenylnaringenin [19]. These developments underscore the necessity to discover water-soluble aromatic PTs with strict flavanone selectivity and enhanced catalytic efficiency for industrial-scale applications.

In this study, we identified an aromatic PT, designated FgPT1, which is capable of catalyzing the prenylation of flavanones from the fungus *F. globosum* through queries of the NCBI and genome databases. Unlike other microbial aromatic PTs that recognize a variety of flavonoid substrates, FgPT1 specifically recognizes six flavanone substrates, yielding a total of seven products, which include one 4′-*O*-prenylated product and six 6-*C*-prenylated products. The catalytic efficiency and substrate affinity of FgPT1 for different flavanones were elucidated through Michaelis–Menten kinetics analysis. Furthermore, we significantly enhanced the catalytic efficiency of FgPT1 to flavanones via site-directed mutagenesis.

## 2. Results and Discussion

### 2.1. Sequence Analysis of FgPT1

**Figure 1:**
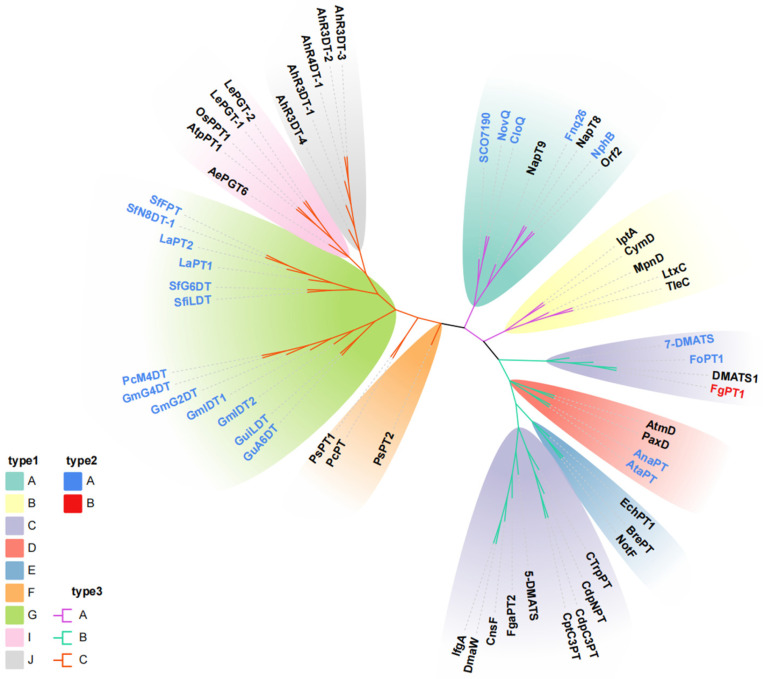
Phylogenetic tree of aromatic prenyltransferases. Type1 color blocks: Represent catalytic substrate skeleton types: A: 1,3,6,8-Tetrahydroxynaphthalene or p-Hydroxybenzoate; B: (-)-Indolactam V or L-Tryptophan; C: L-Tryptophan; D: Complex indole derivatives; E: Tryptophan derivatives; F: Coumarins; G: Flavonoids; I: Propyl 4-hydroxybenzoates; J: Stilbenes. Type 2 color blocks: Blue A: Enzymes characterized to catalyze flavonoids; Red B: Enzymes studied in this work. Type 3 is the line in the middle of the figure, and the different colors of the lines represent the different sources of the enzyme: A (purple) represents that it comes from bacteria; B (green) represents that it comes from fungi; and C (red) represents that it comes from plants. Accession number: AtPPT1, XP_020873525.1; OsPPT1, BAE96574.1; LePGT-2, BAB84122.1; LePGT-1, BAB84123.1; AePGT6, ANC67959.1; SfN8DT-1, BAG12671.1; SfFPT, AHA36633.1; SfiLDT, BAKS2290.1; SfG6DT, BAKS2291.1; GmG2DT, BAW32578.1; GmG4DT, BAH22520.1; GmlDT1, BAW32576.1; GmlDT2, BAW32577.1; PcM4DT, AYV64464.1; LaPT1, AER35706.1; LaPT2, AWK21939.1; GuA6DT, AIT11912.1; GuiLDT, AMR58303.1; PcPT, BAO31627.1; PsPT1, AJW31563.1; PsPT2, AJW31564.1; AhR4DT-1, AQM74172.1; AhR3′DT-1, AQM74173.1; AhR3′DT-2, AQM74174.1; AhR3′DT-3, AQM74175.1; AhR3′DT-3, AQM74176.1; DMATS1, S0EH60.1; NovQ, AAF67510.2; IptA, BAJ07990.1; LtxC, AAT12285.1; CloQ, AAN65239.1; Fnq26, V; FgPT1, KAF5697717.1; IfgA, W6QIM8.1; CTrpPT, ADI60056.1; FgaPT2, AAX08549.1; 5-DMATS, XP_001269817.1; DmaW, C5FTN3.1; CnsF, A0A0A2JWD0.1; CdpNPT, XM_001259404.1; AnaPT, A1DN10; AtaPT, AMB20850.1; BrePT, AFM09725.1; NphB, 7FHF_A; CdpC3PT, XM_001259404.1; FoPT1, XP_031053111.1; MpnD, 4YL7_A; TleC, 4YZK_A; Orf2, BAE00106.1; CptC3PT, XP_001259405.1; NapT9, ABS50462.1; 7-DMATS, EAL92290.2; SCO7190, CAC01590.1; NotF, E0Y3X1.1; EchPT1, ATP76206.1; PaxD, AAK11526.2; AtmD, A9JPE1.2; CymD, 6OS6_A; NapT8, ABS50489.1.

Leveraging our previously characterized FoPT1 sequence as a query, we identified a novel aromatic prenyltransferase FgPT1 (GenBank: KAF5697717.1) in *Fusarium globosum* through homology-guided genome mining, encoding a 426-residue protein with 82% sequence identity to FoPT1. Phylogenetic reconstruction using maximum-likelihood algorithms in MEGA 11 revealed that aromatic PTs cluster into three evolutionarily distinct clades (Figure 1): (i) membrane-bound plant PTs; (ii) fungal soluble PTs; (iii) bacterial soluble PTs, consistent with their taxonomic origins and structural features. Notably, FgPT1 was clustered within the branch of fungal water-soluble PTs that utilize tryptophan and its derivatives as substrates, identifying it as a member of the dimethylallyl tryptophan synthetase (DMATS) family. Members of this family are characterized by broad substrate promiscuity, mediating the prenylation of indole derivatives and acting on diverse aromatic compounds, including flavonoids [20]. AnaPT, which shares 23% sequence identity with FgPT1, has been reported to catalyze the generation of structurally diverse mono-prenylated or bis-prenylated products from various chalcones [21,22]. Similarly, the multifunctional AtaPT modifies diverse flavonoids (daidzein, naringenin, etc.) with variable prenyl chain lengths (C5, C10, C15) [23]. Our previously characterized FoPT1 exhibits a broad substrate scope spanning hesperetin to luteolin [24]. Based on the aforementioned findings, we speculated on FgPT1′s potential to catalyze flavonoid prenylation.

### 2.2. Preparation of FgPT1 and Functional Characterization of Its Prenylated Flavonoids

**Figure 2:**
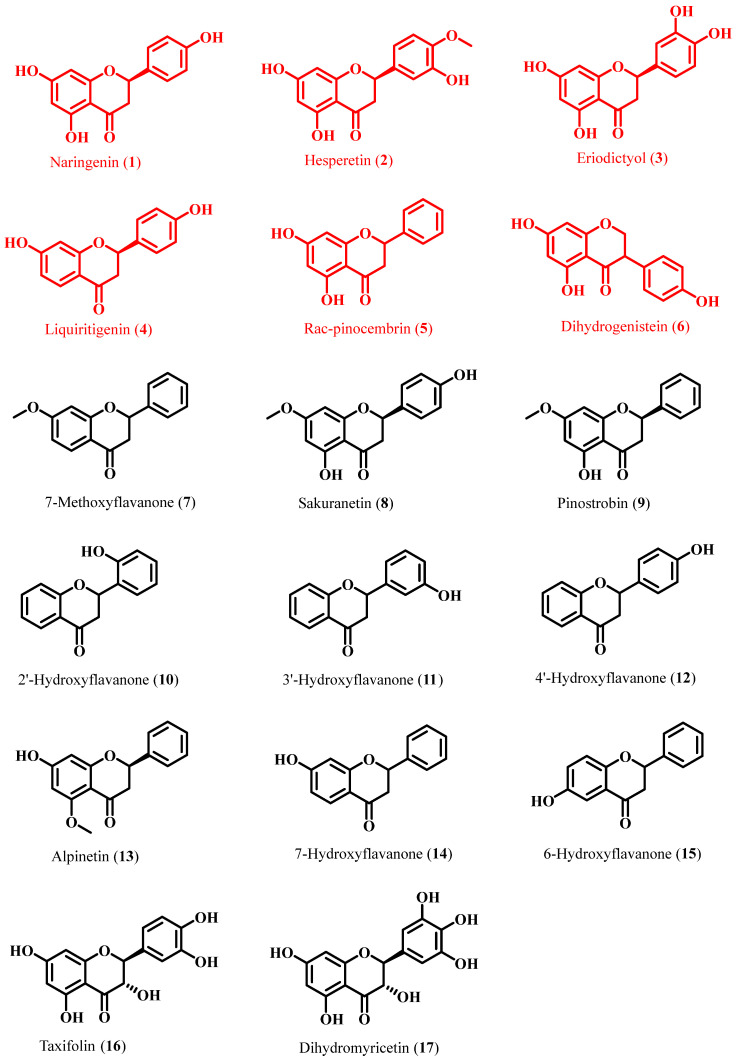
Structure of the substrates of FgPT1 (**1**−**17**).

The FgPT1 gene was cloned into pET-28a for heterologous expression in *E. coli*. Recombinant His*6-tagged FgPT1 (calculated MW: 50.7 kDa) was purified via Ni-NTA affinity chromatography following isopropyl *β*-D-1-thiogalactopyranoside (IPTG) induction (Figure S1). Substrate screening against fifty-six flavonoids (**1**–**56**) using DMAPP as the prenyl donor revealed catalytic activity exclusively toward six flavanones: naringenin (**1**), hesperitin (**2**), eriodictyol (**3**), liquiritigenin (**4**), rac-pinocembrin (**5**), and dihydrogenistein (**6**) (Table 1, Figure 2, and Figure S2). No activity was detected with other flavonoid subclasses (chalcones, flavones, flavonols, isoflavones). This narrow substrate preference starkly contrasts with the broad catalytic promiscuity of characterized microbial aromatic PTs. For instance: AtaPT (*Aspergillus terreus*) catalyzes geranylation of diverse chalcones (2-hydroxychalcone, 3-hydroxychalcone, 4-hydroxychalcone, isoliquiritigenin) and biflavonoids (genistein, putraflavone, amentoflavone, hinokiflavone) [25]; CloQ from *Streptomyces roseochromogenes* exhibits activity toward flavones (4′,7-dihydroxyflavone, luteolin, 4′-hydroxy-7-methoxyflavone), isoflavonoids (equol, daidzein, genistein, 3′-hydroxydaidzein), and flavonoid derivatives (coumestrol) [26]; while Fusarium oxysporum FoPT1 demonstrates versatility across flavones (apigenin, luteolin), flavonols (kaempferol), flavanones (naringenin, hesperetin), and isoflavones (genistein) [24]. The remarkably stringent selectivity of FgPT1 toward flavanone scaffolds positions it as a promising tool enzyme for the specific synthesis of prenylated flavanones.

### 2.3. Isolated Identification and Structural Analysis of Prenylation Products

Preliminary analyses using LC-MS/MS (LCMS-IT-TOF, Shimadzu, Japan) were conducted to identify the enzyme-activated products. Seven prenylated products, namely **1a**, **2a**, **3a**, **4a1**, **4a2**, **5a**, and **6a**, were identified from the incubation mixtures of FgPT1 and DMAPP with substrates **1**, **2**, **3**, **4**, **5**, and **6**, respectively. To further elucidate the structure of the products, the reaction was scaled up, allowing for the isolation and purification of the reaction products, which were subsequently analyzed using NMR.

**Figure 3:**
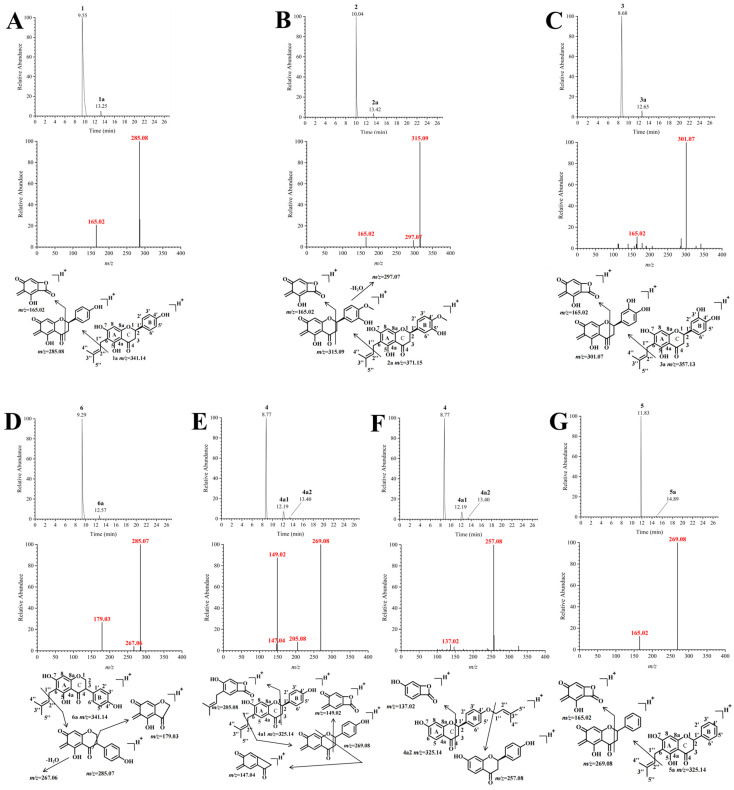
The prenylated products analyzed by UPLC-MS/MS at positive mode. (**A**) Total ion flow diagram of naringenin (**1**) and prenylated naringenin (**1a**), MS2 fragmentation of the prenylated product and the proposed fragmentation pathway of prenylated products. (**B**) Total ion flow diagram of hesperitin (**2**) and prenylated hesperitin (**2a**), MS2 fragmentation of the prenylated product and the proposed fragmentation pathway of prenylated products. (**C**) Total ion flow diagram of eriodictyol (**3**) and prenylated eriodictyol (**3a**), MS2 fragmentation of the prenylated produc and the proposed fragmentation pathway of prenylated products. (**D**) Total ion flow diagram of dihydrogenistein (**6**) and prenylated dihydrogenistein (**6a**), MS2 fragmentation of the prenylated product and the proposed fragmentation pathway of prenylated products. (**E**) Total ion flow diagram of liquiritigenin (**4**) and prenylated liquiritigenin (**4a1**), MS2 fragmentation of the prenylated product and the proposed fragmentation pathway of prenylated products. (**F**) Total ion flow diagram of liquiritigenin (**4**) and prenylated liquiritigenin (**4a2**), MS2 fragmentation of the prenylated product, and the proposed fragmentation pathway of prenylated products, respectively. (**G**) Total ion flow diagram of rac-pinocembrin (**5**) and prenylated rac-pinocembrin (**5a**), MS2 fragmentation of the prenylated product, and the proposed fragmentation pathway of prenylated products, respectively.

The mass spectral results for the prenylated product of naringenin (**1a**) were presented in Figure 3A. The Total Ion Chromatogram (TIC) revealed a sharp product peak at 13.25 min. In the positive ion mode, the parent ion [M + H]^+^ was observed at *m*/*z* 341.14, which aligned with the molecular weight of monoprenylated naringenin. The appearance of the daughter ion [M + H]^+^ at *m*/*z* 285.08 indicated cleavage of the prenyl group between *C*-12″ and *C*-2″, suggesting that the prenylation occurs at the A ring. Additional fragmentation, involving the breakage of the *O*-1 and *C*-2 bonds as well as the *C*-3 and *C*-4 bonds, resulted in the formation of the fragment [M + H]^+^ at *m*/*z* 165.02. The chemical structure of compound 1a was analyzed using NMR, with the chemical shift assignments detailed in Table S1. Characteristic hydrogen signals of the prenyl group were observed at *δ* 3.10 (*H*-1″, d, *J* = 7.1 Hz), *δ* 5.09–5.15 (*H*-2″, brt, *J* = 7.1 Hz), *δ* 1.69 (*H*-4”, s), and *δ* 1.60 (*H*-5″, s). Additionally, *δ* 7.30 (*H*-2′/*H*-6′, d, *J* = 8.6 Hz), *δ* 6.78 (*H*-3′/*H*-5′, d, *J* = 8.6 Hz), and *δ* 1.69 (*H*-4″, s) represented further characteristic signals of the prenyl group, attributed to two equivalent hydrogen groups on the benzene ring (B ring). The signals at *δ* 2.65 (dd, *J* = 17.1 and *J* = 3.1 Hz) and *δ* 3.21 (dd, *J* = 17.1 and *J* = 12.8 Hz) were assigned to *H*-3, while *δ* 5.38 (dd, *J* = 12.8 and *J* = 3.1 Hz) was coupled to *H*-3 and assigned to *H*-2 of the C-ring. The signal at *δ* 5.92 (s) corresponded to the proton signal of the A-ring, with the disappearance of another proton signal from the ring likely due to the substitution of this hydrogen atom by the prenyl side chain. This observation further confirmed that prenylation occurred on the A ring. The *H*-1″ signal (*δ* 3.10, d, *J* = 7.1 Hz) indicated that 1a was a C-prenylated derivative. Moreover, remote coupling of H-1″ (*δ* 3.10) to *C*-5 (*δ* 160.98), *C*-6 (*δ* 107.96), and *C*-7 (*δ* 164.67) was detected using 1H heteronuclear multiple bond correlation (HMBC), thereby confirming that the prenylated moiety was located at *C*-6 (Figures S3–S6). These results indicate that **1a** was identified as 6-*C*-prenyl naringenin.

The mass spectral results for the prenylated product (**2a**) of hesperitin are presented in Figure 3B. The TIC displayed a sharp peak at 13.42 min, corresponding to the product signal (**2a**). In positive ion mode, the product [M + H]^+^ ion was observed at *m*/*z* 371.15, aligning with the molecular weight of monoprenylated hesperitin. The MS2 profile revealed that the principal fragment at *m*/*z* 315.09 results from the cleavage of prenyl *C*-1″ and *C*-2″ between the *O*-1 and *C-*2 bonds, as well as between the *C*-3 and *C*-4 bonds, leading to a further fragment at *m*/*z* 165.02. Additionally, upon losing one water molecule, the fragment at *m*/*z* 315.09 produced a fragment at *m*/*z* 297.07. The presence of these fragment ions substantiated that prenylation occurred at the A-ring. In the NMR (Figure S7), the signals were as follows: *δ* 3.35 (*H*-1″, d, *J* = 7.2 Hz), *δ* 5.22–5.27 (*H*-2″, brt, *J* = 7.2 Hz), *δ* 1.82 (*H*-4”, s), and *δ* 1.76 (*H*-5″, s), which corresponded to the proton signals of the prenyl group. The *H*-1″ signal (*δ* 3.35, d, *J* = 7.2 Hz) indicated that **2a** was a C-prenylated derivative. The HMBC mapping of *H*-1″ (*δ* 3.35) with *C*-5 (*δ* 161.35), *C*-6 (*δ* 107.04), and *C-*7 (*δ* 163.91) exhibited remote coupling relationships, confirming that **2a** is the product of a prenyl unit substitution at *C*-6 (Table S1 and Figures S7–S10). Consequently, **2a** was identified as a 6-*C*-prenyl hesperitin.

The mass spectral results of the prenylated product (**3a**) of eriodictyol were presented in Figure 3C. In the TIC, a sharp peak was observed at 12.65 min, which corresponded to the product peak. The product [M + H]^+^ in positive ion mode was *m*/*z* 357.13, which aligned with the molecular weight of monoprenylated eriodictyol (**3a**). The main fragment observed in the MS2 pattern at *m*/*z* 301.07 represented the parent ion’s prenyl *C*-1″ and *C*-2″ cleavage products, occurring between the C-ring *O*-1 and *C*-2, as well as *C*-3 and *C*-4 bonds. These fragments subsequently underwent further fragmentation and cyclization, resulting in smaller fragments at *m*/*z* 165.02. These fragment ions supported the conclusion that prenylation occurred in the A ring. The carbon and hydrogen spectra of **3a** were obtained using NMR, with the chemical shift values detailed in Table S1: *δ* 3.10 (*H*-1″, d, *J* = 7.4 Hz), *δ* 5.09–5.15 (*H*-2″, brt, *J* = 7.4 Hz), *δ* 1.69 (*H*-4”, s), and *δ* 1.61 (*H*-5″, s), which were characteristic signals of the C-prenyl group (Figures S11–S12). No proton signal was detected at *H*-6, and a single peak without coupling constant was observed at *H*-8 (*δ* 5.92, s), suggesting that the prenyl chain was attached to *C*-6. Consequently, **3a** was identified as 6-*C*-prenyl eriodictyol.

The mass spectral results for the prenylated product (**6a**) of dihydrogenistein were illustrated in Figure 3D. The TIC revealed a distinct product peak at 12.57 min. In positive ion mode, the observed product ion [M + H]^+^ was at *m*/*z* 341.14, aligning with the molecular weight of prenyl dihydrogenistein. The three fragment ions at *m*/*z* 285.07, *m*/*z* 267.06, and *m*/*z* 179.03 noted in MS2 were believed to arise from the cleavage of the parent ions prenyl *C*-1″ and *C*-2″, resulting in the loss of one molecule of water during the fragmentation process. This cleavage occurred at the C ring and led to autocyclization, as depicted in Figure 3 (D). These fragment ions suggested that prenylation took place on the A ring. The NMR spectra of **6a** were presented in Figures S13–S16, with their corresponding chemical shift assignments listed in Table S1. Characteristic signals for the prenyl group included *δ* 3.34 (*H*-1′, d, *J* = 7.2 Hz), *δ* 5.22–5.27 (*H*-2″, brt, *J* = 7.2 Hz), *δ* 1.81 (*H*-4”, s), and *δ* 1.76 (*H*-5″, s). The typical chemical shift for *H*-1″ (*δ* 3.34, d, *J* = 7.2 Hz) further supported the conclusion that 6a was a C-prenylated derivative. Additionally, the HMBC correlations from *H*-1″ (*δ* 3.34) to *C*-5 (*δ* 161.71), *C*-6 (*δ* 106.96), and *C*-7 (*δ* 163.38) indicated that the prenyl substitution occurred specifically at the *C*-6 position. Consequently, **6a** was identified as 6-*C*-prenyl dihydrogenistein.

The mass spectral results of the prenylated product (**4a**) derived from liquiritigenin were presented in Figure 3E. The TIC revealed distinct product peaks **4a1** and **4a2** at retention times of 12.19 min and 13.40 min, respectively, with **4a1** as the primary product, exhibiting a peak height ratio of 4:1 relative to **4a2**. Both products exhibited an *m*/*z* of 325.14 in the positive ion mode, consistent with the molecular weight of monoprenylated liquiritigenin. The fragmentation pattern of **4a1** closely resembled that of previously characterized 6-*C* prenyl derivatives, including naringenin, hesperidin, eriodictyol, and dihydrogenistein (Figure 3E). The MS2 fragmentation analysis revealed ions at *m*/*z* 269.08, *m*/*z* 149.02, and *m*/*z* 205.08, which were attributed to the cleavage of the prenyl side chain between *C*-1″ and *C*-2″. Furthermore, the cleavage of the prenyl side chain in the C-ring led to the formation of both the cleavage product and the parent ion through autocyclization. A minor fragment at *m*/*z* 147.04 was also observed, likely resulting from the cyclization of the primary fragment *m*/*z* 269.08 between *O*-1 and *C*-8a, as well as between *C*-2 and *C*-3. These MS findings indicated that the prenyl side chain of 4a1 was connected to the A ring. The NMR profile for 4a1 was illustrated in Figures S17 and S18, and its chemical shift assignments are detailed in Table S1. Characteristic signals for the prenyl group included *δ* 3.16 (*H*-1″, d, *J* = 7.4 Hz), *δ* 5.21–5.29 (*H*-2″, brt, *J* = 7.4 Hz), *δ* 1.70 (*H*-4”, s), and *δ* 1.65 (*H*-5″, s). The chemical shift value of *H*-1″ (*δ* 3.16, d, *J* = 7.4 Hz) further supported the identification of **4a1** as a C-prenylated derivative. No proton signal was detected at *H*-6, and a single peak without coupling constant was observed at *H*-8 (*δ* 6.37, s), suggesting that the prenyl chain was attached to *C*-6. Consequently, **4a1** was identified as 6-*C* prenyl liquiritigenin.

The MS2 of compound **4a2** revealed a primary fragment at *m*/*z* 257.08, which was presumably generated by the detachment of the prenyl side chain from the parent ion—a phenomenon commonly observed in the secondary fragments of various O-prenylation products [27]. The C-ring of the central fragment at *m*/*z* 257.08 underwent cleavage and autocyclization, resulting in the formation of the fragment at *m*/*z* 137.02 (Figure 3F). The MS data suggested that **4a2** was like an O-prenylation product; however, the specific phenolic hydroxyl group at which the prenyl substitution occurred remained ambiguous. To further elucidate the structure of **4a2**, NMR spectroscopy was employed, with pertinent results illustrated in Figures S19–S22 and the corresponding chemical shifts are listed in Table S1. The *H*-1″ signal (*δ* 4.53, d, *J* = 6.8 Hz) provided additional evidence supporting the classification of **4a2** as an O-prenylated derivative. HMBC correlations from *H*-1″ (*δ* 4.53) to *C*-2″ (*δ* 119.41), *C*-3” (*δ* 138.59), and *C*-4′ (*δ* 159.27) confirmed that the prenyl group was attached to the *C*-4′ phenolic hydroxyl group. Consequently, **4a2** was identified as 4′-*O*-prenyl liquiritigenin.

The mass spectral results of the prenylated product (**5a**) derived from rac-pinocembrin were presented in Figure 3G. A sharp peak observed at 14.89 min in the TIC corresponds to the product signal. The product [M + H]^+^ in positive ion mode was measured at *m*/*z* 325.14, aligning with the molecular weight of monoprenyl rac-pinocembrin. Fragment ions at *m*/*z* 269.08 and *m*/*z* 165.02 in MS2 were presumed to result from cleavage between the parent ions at prenyl *C*-1″ and *C*-2″. This cleavage was hypothesized to occur by breaking the *O*-1 to *C*-2 bond and the *C*-3 to *C*-4 bond in the C ring, followed by cyclization. The secondary fragmentation suggested that the prenyl side chain **5a** was likely attached to the C of the A ring. Due to the insufficient yield of **5a** in the scaled-up reaction system, there were not enough products available for NMR detection, preventing a definitive clarification of its structure. This aspect will be addressed in our future work.

Through the catalysis of flavanones by FgPT1, we obtained six C-prenylated products and one O-prenylated product. Unlike plant membrane-bound aromatic PTs and the previously discussed FoPT1, which exhibited high regioselectivity, FgPT1 preferentially catalyzed prenylation at the *C*-6 position of flavanones while also mediating 4′-*O*-prenylation reactions. This regioselectivity suggested that FgPT1 could synthesize structurally diverse prenylated flavanones.

### 2.4. Characterization of the Catalytic Properties of FgPT1

#### 2.4.1. Metal-Cation Dependence of FgPT1

**Figure 4:**
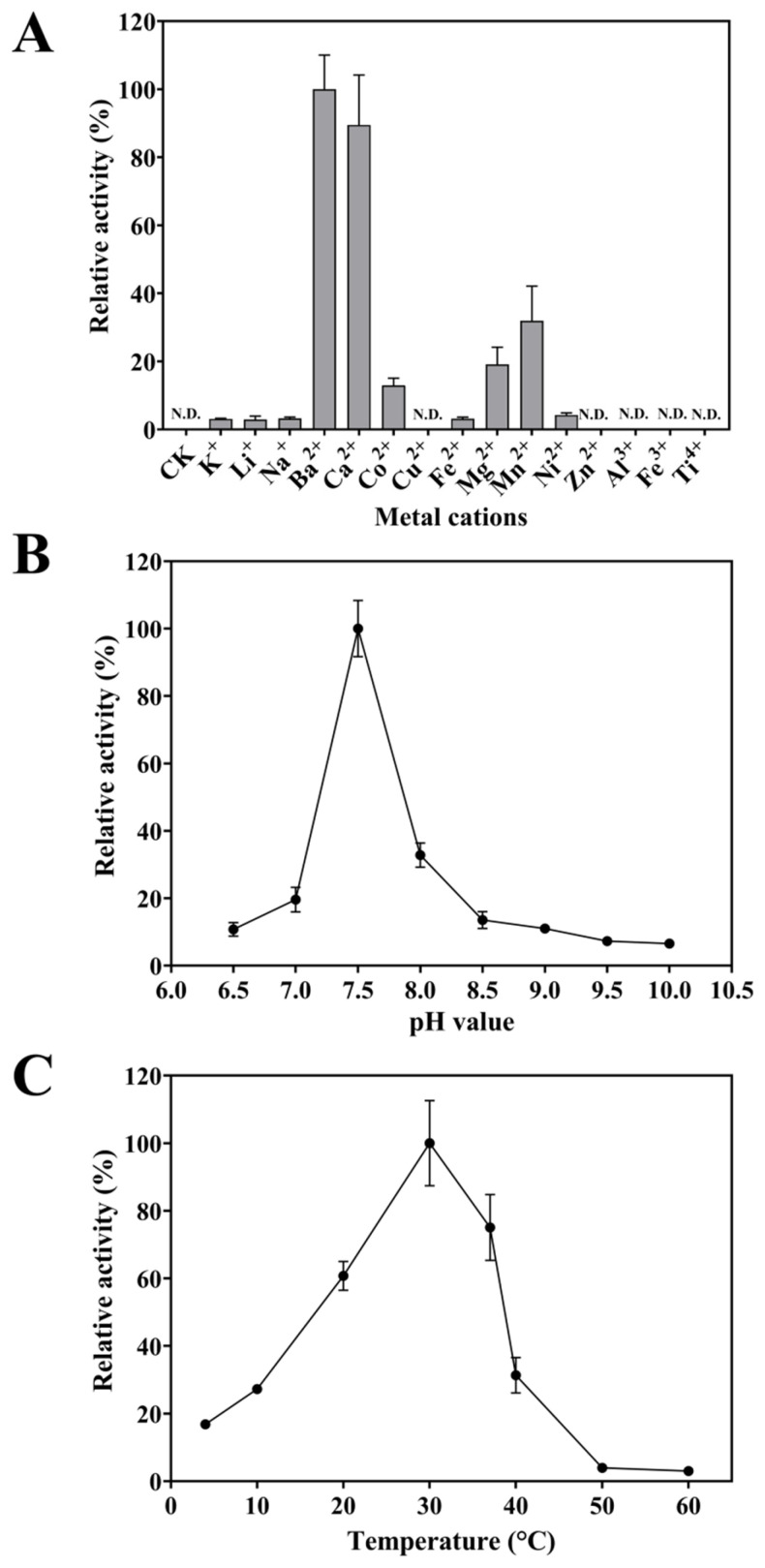
The enzymatic properties of FgPT1. (**A**) The effects of metal ions when incubated with liquiritigenin. Note that CK represents the absence of metal ions, and N.D. represents not detected activity. (**B**) The effects of environmental pH when incubated with liquiritigenin. (**C**) The effects of environmental temperature when incubation with liquiritigenin.

Distinct cofactor requirements typically accompanied the functional diversification of microbial PTs. To investigate the metal ion dependency of FgPT1, 15 metal cations with different valence states (K^+^, Li^+^, Na^+^, Ba^2+^, Ca^2+^, Co^2+^, Cu^2+^, Fe^2+^, Mg^2+^, Mn^2+^, Ni^2+^, Zn^2+^, Al^3+^, Fe^3+^, and Ti^4+^) were added with liquiritigenin as substrate to test conversation efficiency of FgPT1 to liquiritigenin, and no metal cations were added as the control. The control group did not detect any product, indicating that FgPT1 is dependent on metal cations to function. Among the metals tested, the catalytic activity of FgPT1 ranked in descending order as follows: Ba^2+^ > Ca^2+^ > Mn^2+^ > Mg^2+^ > Co^2+^ > Ni^2+^ > Na^+^ > Fe^2+^ > Li^+^ > K^+^. Adding the remaining metal ions did not show any catalytic activity from FgPT1 (Figure 4A). Most PTs of microbial origin were typically non-dependent on metal ions. For instance, neither the presence nor absence of Ca^2+^ influenced the prenylation activity of FtmPT2 on 12,13-dihydroxyfumitremorgin C [28]. However, it has also been reported that although some PTs do not rely on metal ions to function, their introduction can greatly improve the reaction efficiency. Specifically, the exogenous addition of 5 mM Mg^2+^ boosted the catalytic activity of FtmPT1 and CdpNPT by 40% and 76%, respectively [29,30]. Consistent with our findings, Orf2 [31] and FoPT1 [24] have also been identified as metal ion-dependent aromatic PTs of microbial origin.

#### 2.4.2. Optimum Reaction PH of FgPT1

The pH values profoundly influence the activity of aromatic PTs, directly modulating their catalytic efficiency. To investigate the effect of varying pH levels on the activity of FgPT1, a buffer solution of 100 mM Tris-HCl was prepared at pH values of 6.5, 7.0, 7.5, 8.0, 8.5, 9.0, 9.5, and 10.0. In vitro experiments used liquiritigenin as the substrate and Ba^2+^ as an essential divalent cation activator. The results in Figure 4B indicate that the optimal reaction pH for FgPT1 was 7.5. The systematic evaluation demonstrated taxon-specific adaptations: FgPT1 exhibited a pH optimum (pH 7.5) comparable to that of FoPT1 (pH 7.4) [24] from the same *Fusarium* genus, suggesting evolutionary conservation of pH adaptation within this fungal lineage. In contrast, the bacterial prenyltransferase ShFPT (*Streptomyces* sp.) demonstrated maximal activity at pH 6.0 while maintaining >65% of peak efficiency across a broad pH range (5.5–7.5) [32]. Intriguingly, biochemical characterization of the silent terpene synthase AaTPS from *Alternaria alternata* TPF6 revealed pH-dependent functional plasticity—this enzyme acquired promiscuous aromatic prenyltransferase activity under alkaline conditions, with maximal activity observed at pH 8.6 [33]. These diverse pH optima, spanning acidic to alkaline regimes (pH 6.0–8.6), underscore the critical need for taxon-specific pH optimization when employing aromatic prenyltransferases as biocatalysts, particularly in bioprocesses requiring robustness under non-native reaction conditions.

#### 2.4.3. Optimum Reaction Temperatures of FgPT1

Temperature is critical for enzyme activity. To assess the impact of temperature on FgPT1 activity, Tris-HCl at pH = 7.5 was employed as the buffer solution, with liquiritigenin as the substrate and Ba^2+^ as an essential divalent cation activator. In vitro experiments were performed at temperatures of 4 °C, 10 °C, 20 °C, 30 °C, 37 °C, 40 °C, 50 °C, and 60 °C. As illustrated in Figure 4C, demonstrated that the optimal reaction temperature for FgPT1 is 30 °C. In contrast, the bacterial prenyltransferase ShFPT exhibited maximal activity at a significantly higher temperature of 50 °C in 50 mM citrate buffer (pH 6.0) [32]. Notably, the thermal profile of FgPT1 aligns more closely with that of the fungal AaTPS, which demonstrated optimal enzymatic activity at 28 °C under comparable assay conditions [33]. These temperature-dependent variations highlight the necessity of tailoring reaction parameters to individual enzyme characteristics.

### 2.5. Kinetic Parameters of the FgPT1 Reaction of Substrates in the Presence of DMAPP

To investigate the catalytic efficiency of FgPT1, we measured the steady-state kinetic parameters (*k_cat_* and *K_M_*) for six flavanones: naringenin, hesperetin, eriodictyol, liquiritigenin, rac-pinocembrin, and dihydrogenistein with saturating DMAPP (1 mM) (Table 2; Figure S23). The kinetic analyses demonstrated significant substrate selectivity, with naringenin identified as the optimal substrate, exhibiting *k_cat_*/*K_M_* of 61.92 s^−1^ M^−1^ for FgPT1. Conversely, FgPT1 displayed the lowest catalytic efficiency for liquiritigenin, with a *k_cat_*/*K_M_* value of 1.18 s^−1^ M^−1^, indicating a 52-fold difference in substrate affinity. Enzymes frequently exhibit substantial differences in affinity for similar types of flavonoids; for instance, AtaPT displayed a 90-fold difference in affinity for various chalcone compounds, while AnaPT exhibited an even significant disparity of 1591-fold [22]. Catalytic efficiency comparisons revealed substrate preferences among aromatic PTs. FgPT1 exhibited a *k_cat_*/*K_M_* value of 61.92 s^−1^ M^−1^ for naringenin, slightly surpassing the 53.38 s^−1^ M^−1^ reported for *Fusarium oxysporum* FoPT1 with the same substrate. However, FgPT1 demonstrated substantially lower efficiency toward hesperetin (3.40 s^−1^ M^−1^) compared to FoPT1 (22.13 s^−1^ M^−1^) [24]. For other flavanones, FgPT1 showed comparable efficiencies: hesperetin (3.40 s^−1^ M^−1^), eriodictyol (6.09 s^−1^ M^−1^), rac-pinocembrin (5.00 s^−1^ M^−1^), and dihydrogenistein (2.14 s^−1^ M^−1^). AnaPT displayed moderate activity toward eriodictyol (13.4 s^−1^ M^−1^), 7-hydroxyflavanone (8.8 s^−1^ M^−1^), silibinin (9.4 s^−1^ M^−1^), genistein (8.2 s^−1^ M^−1^), and apigenin (16.4 s^−1^ M^−1^), while 7-DMATS exhibited enhanced catalytic turnover for genistein (171 s^−1^ M^−1^) compared to naringenin (23 s^−1^ M^−1^) and hesperetin (24 s^−1^ M^−1^) [21]. Notably, AtaPT achieved remarkable efficiencies with biflavonoids—putraflavone (150 s^−1^ M^−1^), amentoflavone (71.4 s^−1^ M^−1^), and hinokiflavone (61.1 s^−1^ M^−1^) [25].

### 2.6. Structural Requirements of FgPT1 for Flavanone Substrates

This study systematically analyzed seventeen flavanones to elucidate structure–activity relationships in FgPT1-catalyzed flavanone prenylation. Catalytically competent substrates (**1**–**6**) uniformly retained a *C*7-OH group on the A-ring. Methoxy substitution (**7**–**9**) or *C*7-OH deletion (**10**–**12**) abolished activity, consistent with prior evidence demonstrating hydrogen-bond interactions between A-ring *C*7-OH/B-ring *C*4′-OH and catalytic pocket residues during substrate recognition [34]. Compounds **13** (A-ring *C*-5 methoxylation) and **14** were not catalyzed either, suggesting that the presence of the *C*-7 hydroxyl group alone is insufficient to drive the reaction. The *k_cat_*/*K_M_* value of naringenin was found to be 52-fold more significant than that of liquiritigenin and 28-fold more significant than that of pinocembrin, implying that both the A-ring *C*-5 hydroxyl and the B-ring *C*-4 hydroxyl significantly enhance the conversion of flavanones [35]. Comparative kinetic analysis revealed significant electronic and steric constraints: Eriodictyol (*C*5′-OH substitution) exhibited a 91% reduction in catalytic efficiency, while dihydrogenistein (B-ring at *C*3) showed 12-fold lower activity than naringenin (B-ring at *C*2). Furthermore, flavanone alcohols **16**–**17** (*C*3-OH substitution) were inactive, highlighting the detrimental effect of C-ring hydroxylation. These positional preferences align with SfFPT’s reliance on A-ring C7/5-OH groups for prenylation site activation [14]. Structural characterization of FgPT1 products confirmed exclusive *C*6-prenylation. The introduction of hydroxyl groups at the *C*-5 and *C*-7 positions enhances the availability of neighboring electrons to the *C*-6 of the prenyl group. Similarly, the electrons from the *OH*-4′ group can be transferred to *C*-6 through the extensive conjugated ring system, thereby increasing the electronegativity of this site [36]. This enhanced electronegativity facilitates the electrophilic attack on *C*-6 by positively charged alkyl side-chain carbon ions.

### 2.7. Structural Insights into FgPT1-Substrate Interactions from Homology Modeling and Docking

**Figure 5:**
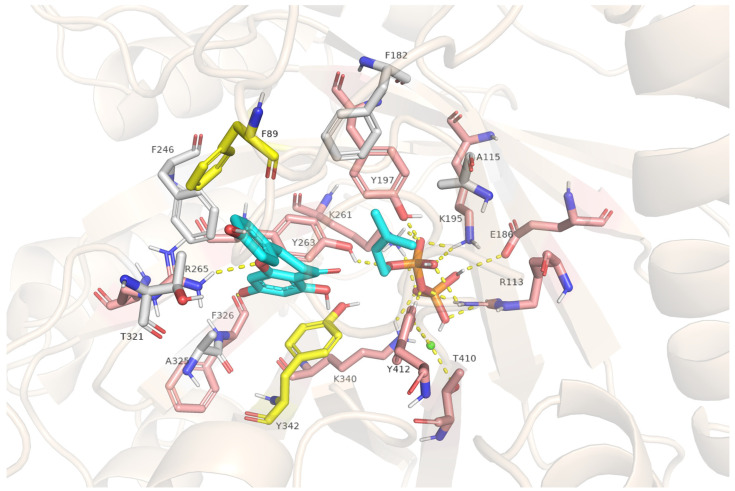
The docking model of FgPT1, DMAPP, naringenin, and Ba^2+^.

To gain insight into the active center of FgPT1, homology modeling was performed by the high sequence identity (89%) with DMATS1 from *Fusarium fujikuroi*. Using the DMATS1 crystal structure bound to DMSPP/tyrosine (PDB: 8DAY) [37]. The result generated an FgPT1 model revealing a conserved *β*-barrel core comprising 10 antiparallel *β*-strands enveloped by 11 *α*-helices (Figure 5). This configuration forms the signature ABBA fold of soluble aromatic PTs, with the central hydrophobic cavity essential for substrate docking [31,38,39,40]. Molecular docking results revealed that the hydrophobic environment created by residues F246, Y263, T321, and A325 facilitated the localization of naringenin. Studies have revealed that π-π stacking interactions and hydrogen bonds between amino acid residues and the substrate significantly enhance substrate stabilization [41]. The B-ring and A-ring of naringenin form π-π stacking interactions with residues F89 and Y342, respectively. Furthermore, hydrogen bonds between the *O*-1 atom of the C-ring and R265, as well as between the 7-hydroxyl group of the A-ring and F326, further stabilize the binding conformation of naringenin within the enzyme’s active site. And Ba^2+^ formed hydrogen bonds with T410 and Y412. Metal ions usually play an important role in stabilizing the binding of phosphate groups and in promoting the formation and cyclization of key intermediate carbon cations [42]. For the prenyl donor DMAPP, its alkyl chain was anchored by hydrophobic residues A115, F182, and Y197, while the pyrophosphate moiety interacts with R113, E186, K195, Y197, K261, Y263, K340, and Y412 via hydrogen bonding. Positively charged residues (R113, K195, K261, K340) likely drive charge polarization to generate a dimethylallyl carbocation analogous to Friedel–Crafts electrophilic aromatic substitution [43]. Spatial proximity between the carbocation and naringenin’s A-ring *C*6 facilitates regioselective electrophilic attack, consistent with the observed *C*6-prenylation specificity.

Considering the high conservation of these residues (except A325) in both DMATS1 and FoPT1, we focused on the divergent amino acids in the *β*-barrel region, including A325.

### 2.8. Impact of Site-Directed Mutagenesis on FgPT1 Catalytic Activity

**Figure 6:**
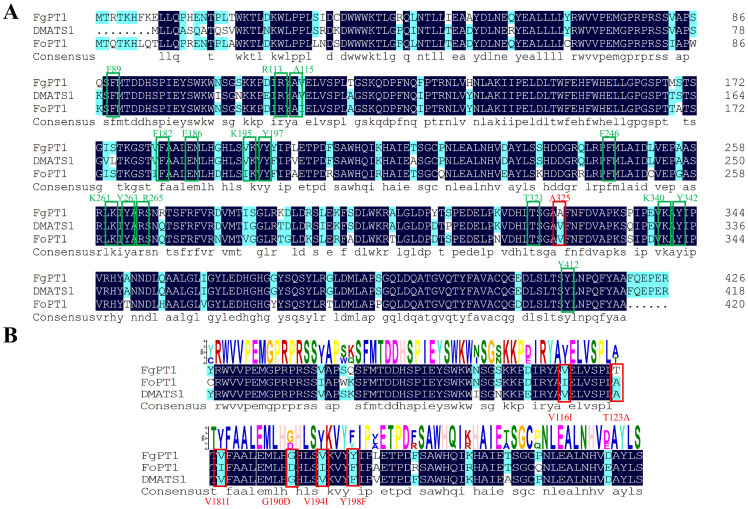
Sequence alignment of FgPT1, FoPT1, and DMATS1 from *Fusarium*. (**A**) Active sites in the docking model of FgPT1 with DMAPP, naringenin, and Ba^2+^ (Green frames represent conservative amino acids; Red frames represent non-conservative amino acids). (**B**) Two conserved gene clusters of DMATS-type prenyltransferase genes. Mutants V116I, T123A, V181I, G190D, V194I, and Y198F are boxed in red.

**Figure 7:**
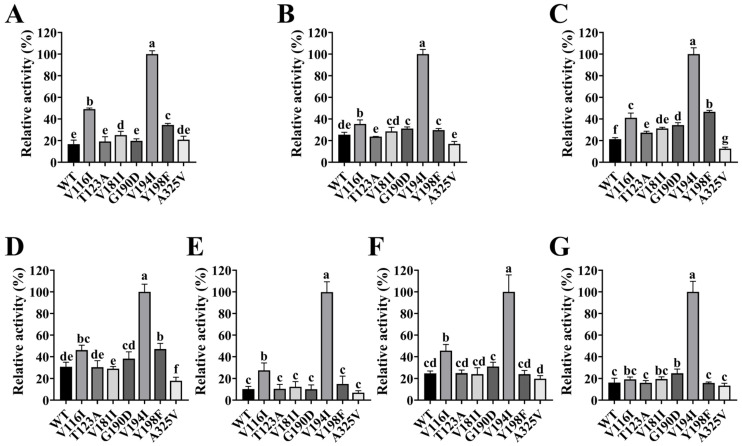
Relative activity of the wild-type and mutants to the prenylated product of the substrates (**1**–**6**). (**A**) 6-*C*-prenyl naringenin (**1a**). (**B**) 6-*C*-prenyl hesperitin (**2a**), (**C**) 6-*C*-prenyl eriodictyol (**3a**), (**D**) 6-*C*-prenyl liquiritigenin (**4a1**), (**E**) 4′-*O*-prenyl liquiritigenin (**4a2**), (**F**) 6-*C*-prenylrac-pinocembrin (**5a**), (**G**) 6-*C*-prenyl dihydrogenistein (**6a**). Different letters indicate statistical significance, determined by one-way ANOVA (*p* < 0.05). Data are presented as mean ± SD (*n* = 3).

Comparative sequence analysis of *F. fujikuroi* DMATS1, *F. oxysporum* FoPT1, and FgPT1 (Figure 6A) identified seven divergent residues (A325, V116, T123, V181, G190, V194, Y198) within the *β*-barrel domain. Site-directed mutagenesis generated seven structural variants (A325V, V116I, T123A, V181I, G190D, V194I, Y198F) engineered according to phylogenetic conservation (Figure 6B). Functional characterization of these mutants against six flavanones—naringenin, hesperetin, eriodictyol, liquiritigenin, rac-pinocembrin, and dihydrogenistein—demonstrated differential catalytic profiles (Figure 7 and Figure S24).

**Figure 8:**
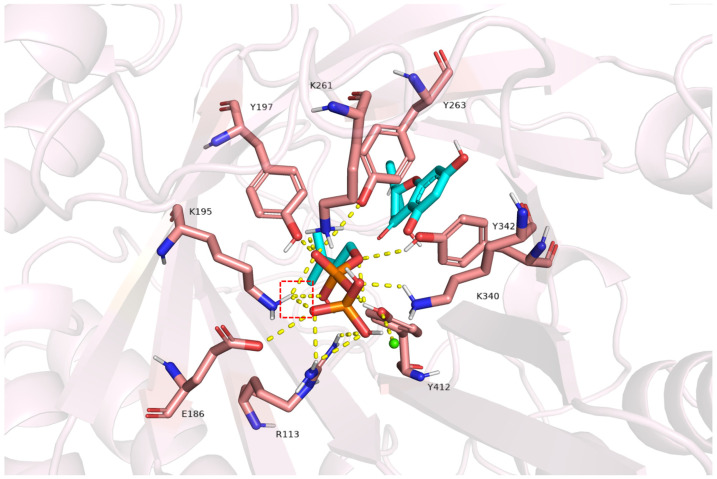
The docking model of V194I mutant. The red box shows the hydrogen bonding connection between K195 and DMAPP.

The A325V mutant exhibited reduced catalytic activity (2.9–51.5% decline across substrates **1**–**6** vs. wild type), likely due to steric hindrance from its bulkier hydrophobic side chain. Molecular docking revealed that the additional methyl groups at position 325 increased the F326-naringenin distance from 2.7 Å to 3.3 Å (Figure S25), weakening hydrogen bonding and mispositioning the substrate [44,45]. Mutants T123A, G190D, and V181I showed negligible activity changes, whereas V116I displayed significantly enhanced catalysis—notably a threefold increase in *k_ca__t_*/*K_M_* for naringenin. Research by Köllner et al. and Bertsch et al. suggested that amino acids adjacent to the active site played a crucial role in shaping its geometry and modulating catalytic efficiency [46,47]. In this study, the side chain of residue 116 was situated between the *β*-folding barrel and the *α*-helix. The mutated isoleucine residue had a longer side chain than the original valine. Structural analysis suggests that the elongated isoleucine side chain at position 116 repositions neighboring A115, improving hydrophobic packing with the DMAPP alkyl chain. Such enhanced substrate-donor interactions align with prior reports that optimized hydrophobic interfaces boost enzymatic affinity and turnover [48]. Surprisingly, the V194I mutant exhibited a significant increase in catalytic activity for six substrates when compared to the wild type, demonstrating an almost nine-fold enhancement in the conversion of naringenin. This observation suggested that the V194I mutation plays a crucial role in substrate binding. Similar to the case of residue 116, residue 194 was situated between the *β*-folding barrel and the *α*-helix, where valine was substituted with isoleucine. This substitution results in an elongation of the side chain, which enhances hydrogen bonding interactions between residue K195 and its donor, increasing the number of hydrogen bonds from two to three (Figure 8). Notably, this included interactions between the added hydrogen and the oxygen on the first phosphate of DMAPP. Concurrently, a new hydrogen bond was formed between pyrophosphate and residue Y342. Establishing these new hydrogen bonds between the enzyme and the substrate can significantly enhance catalytic efficiency [49,50,51]. Furthermore, since K195 was also involved in the formation of the dimethylallyl carbon cation, the distance between the side-chain amino group of K195 and the donor pyrophosphate was reduced following the mutation, thereby strengthening the ionic bond between them. This enhancement favored the dissociation of pyrophosphate and the formation of the carbon cation. Therefore, it was hypothesized that the simultaneous enhancement in hydrogen and ionic bonding between the active center and the donor was a key factor contributing to the marked increase in conversion efficiency observed in the V194I mutant.

## 3. Materials and Methods

### 3.1. Chemical Reagents

Naringenin chalcone, alpinetin, flavanone, eriodictyol, and sakuranetin were procured from Alfa Aesar (Shanghai, China), while 7-hydroxyflavanone was obtained from Ron (Shanghai, China). Additional flavonoid substrates and kanamycin, IPTG, NaCl, glycerol, imidazole, KCl, LiCl, NaCl, BaCl_2_, CaCl_2_, CoCl_2_, CuCl_2_, FeCl_2_, MgCl_2_, MnCl_2_, NiCl_2_, ZnCl_2_, AlCl_3_, FeCl_3_, and Ti(SO_4_)_2_ were sourced from Aladdin (Shanghai, China). Agilent Technologies Ltd. (Beijing, China) acquired HPLC-grade methanol and acetonitrile. Dimethylallyl pyrophosphate (DMAPP) was synthesized chemically following previously established methods [15]. The components of the lysogeny broth (LB) medium, including tryptone, yeast extract, and sodium chloride, were purchased from Sangon Biotech (Shanghai, China).

### 3.2. Synthesis of the FgPT1 Gene

The gene information for FgPT1 (Accession number: KAF5697717.1) was retrieved from the NCBI database, and chemical synthesis was performed by Sangon Biotech in Shanghai, China. The FgPT1 gene was subsequently cloned and ligated into the pET-28a vector, resulting in the construction of the pET-28a-FgPT1 vector, which includes a His*6 tag at the N-terminus.

### 3.3. Expression and Purification of the FgPT1 Gene

The recombinant plasmid pET-28a-FgPT1 was transformed into BL21 (DE3) competent cells following the manufacturer’s protocol for BL21 (DE3) Receptor Cells (EC1002S, Weidi Bio, Shanghai, China). The transformants were evenly spread onto a solid LB medium containing kanamycin using the spread plate method and cultured overnight at 37 °C. Individual colonies were then selected and inoculated into 50 mL of LB liquid medium supplemented with kanamycin, followed by overnight incubation at 37 °C with shaking at 200 rpm to prepare a seed culture. Subsequently, 2% of this seed culture was transferred into 1 L of LB liquid medium with kanamycin and incubated at 37 °C with shaking at 200 rpm until the optical density at 600 nm (OD_600_) reached 0.6–0.8. The culture was then cooled in an ice water bath for 30 min. IPTG was added to a final concentration of 1 mM, and the cultures were further incubated at 16 °C with shaking at 200 rpm for 18 h to induce FgPT1 protein expression. Finally, cells were harvested by centrifugation at 10,000× *g* for 5 min at 4 °C.

Cells were resuspended in a lysis buffer of 50 mM Tris-HCl (pH = 7.5), 100 mM NaCl, 5% glycerol (*v*/*v*), and 5 mM imidazole. The cells were subjected to sonication on the ice at 45% power, with a cycle of 3 s on and 3 s off for 40 min, utilizing an ultrasonic cell breaker (NY-JY92-IIN, Enyi, Changzhou, China). The resulting precipitate was removed via centrifugation at 12,000× *g* for 15 min at 4 °C. The supernatant was then passed through a Ni-NTA protein purification column (Qiagen, Hilden, Germany). Heteroproteins were eliminated using a protein wash buffer containing 50 mM Tris-HCl (pH = 7.5), 100 mM NaCl, 5% glycerol, and 40 mM imidazole. The target protein was subsequently eluted using a 50 mM Tris-HCl (pH = 7.5) buffer, 100 mM NaCl, 5% glycerol (*v*/*v*), and 250 mM imidazole. The elution buffer containing the target proteins was concentrated with a 30 kDa centrifugal filter (Amicon Ultra-15, Millipore, Shanghai, China), and a protein desalting buffer composed of 50 mM Tris-HCl (pH = 7.5), 100 mM NaCl, 10% glycerol, and 2 mM dithiothreitol was employed for desalting the concentrated protein. Protein concentration was determined using an ultra-micro spectrophotometer (Colibri, Berthold, Beijing, China). To assess protein purity, Sodium dodecyl sulfate-polyacrylamide gel electrophoresis was performed. Purified FgPT1 was stored at −80 °C.

### 3.4. Measurement of FgPT1 Activity

Enzyme activity was assessed in a reaction system consisting of 150 μL, which included 50 mM Tris-HCl (pH = 7.5), 500 μM flavonoid substrate, 10 mM metal ions, 1 mM DMAPP, and 130 μg of recombinant FgPT1. Following a 5 h incubation at 37 °C, the products were extracted using ethyl acetate (3 × 150 μL). The organic phase was then evaporated to dryness using a vacuum centrifugal concentrator (ZL3-1K, Kecheng, Hunan, China). Subsequently, the residue was redissolved in 150 μL of acetonitrile, filtered to eliminate impurities, and analyzed via HPLC.

To investigate the influence of metal cations on the catalytic activity of FgPT1, reactions were supplemented with various salts, including KCl, LiCl, NaCl, BaCl_2_, CaCl_2_, CoCl_2_, CuCl_2_, FeCl_2_, MgCl_2_, MnCl_2_, NiCl_2_, ZnCl_2_, AlCl_3_, FeCl_3_, and Ti(SO_4_)_2_, all at a final concentration of 10 mM. A reaction devoid of added metal ions served as a control. To assess the effect of pH on FgPT1 activity, Tris-HCl buffer was utilized at pH values of 6.5, 7.0, 7.5, 8.0, 8.5, 9.0, 9.5, and 10.0. The impact of incubation temperature on FgPT1 activity was evaluated by conducting reactions at various temperatures: 4 °C, 10 °C, 20 °C, 30 °C, 37 °C, 40 °C, 50 °C, and 60 °C. All measurements were carried out in triplicate.

### 3.5. Identification and Preparation of Products

HPLC analysis used a Hitachi Chromaster (Tokyo, Japan) with an Agilent Infinity Lab Poroshell 120 SB-C18 column (3.0 × 100 mm, 2.7 μm, 600 bar). The flow rate was maintained at 0.5 mL min^−1^, the column temperature was set to 30 °C, and the injection volume was 10 μL. The mobile phase comprised solvent A (ultrapure water) and solvent B (acetonitrile). The elution procedure for HPLC was as follows: 0–5 min, 10% B; 5–8 min, 10–35% B; 8–25 min, 35–70% B; 25–26 min, 70–100% B; 26–30 min, 100%; 30–31 min, 100–10% B; and 31–37 min, 10% B. UPLC-MS/MS analysis was performed using an ion trap-time-of-flight mass spectrometer (LCMS-IT-TOF, Shimadzu, Japan) with an ACQUITY UPLC HSS-C18 column (2.1 × 150 mm, 1.8 μm). The mobile phase consisted of solvent A (ultrapure water containing 0.5% formic acid) and solvent B (acetonitrile). The elution program for UPLC-MS/MS analysis was as follows: 0–3 min, 10% B; 3–16 min, 10–95% B; 16–20 min, 95% B; 20–21 min, 95–10% B; and 21–27 min, 10% B. In positive ion mode, analyte ionization was achieved using an electrospray ionization interface with a collision voltage set at 50 eV. The mass scan range was configured to *m*/*z* 100–1000.

The products were prepared using a semi-preparative HPLC system (LC-20AP, Shimadzu, Japan) equipped with an Agilent ZORBAX SB-C18 column (9.4 × 150 mm, 5 μm). The flow rate was set at 4 mL min^−1^, and the injection volume was 0.5 mL. The mobile phase comprised solvents A (ultrapure water) and B (methanol). The elution procedure for the semi-preparative HPLC analysis was as follows: for naringenin, 0–2 min at 10% B, 2–6 min at 45% B, 6–13 min at 51% B, 13–33 min at 70% B, 33–43 min at 90% B, and 43–50 min at 100% B; for hesperitin, 0–2 min at 10% B, 2–6 min at 51% B, 6–19 min at 71% B, and 19–23 min at 100% B; for eriodictyol, 0–2 min at 10% B, 2–6 min at 45% B, 6–13 min at 51% B, 13–33 min at 70% B, 33–43 min at 90% B, and 43–50 min at 100% B; for dihydrogenistein, 0–2 min at 10% B, 2–6 min at 45% B, 6–13 min at 51% B, 13–33 min at 70% B, 33–43 min at 90% B, and 43–50 min at 100% B; for liquiritigenin, 0–2 min at 10% B, 2–8 min at 51% B, 8–21 min at 65% B, 21–29 min at 70% B, 29–37 min at 85% B, and 37–42 min at 100% B; and for rac-pinocembrin, 0–1 min at 10% B, 2–11 min at 70% B, 11–21 min at 85% B, and 21–26 min at 100% B. One-dimensional and two-dimensional NMR analyses were performed using a bruker avance neo 600 MHz spectrometer, and the resulting maps were processed with MestReNova 15.0.0. Chemical shifts were calibrated concerning the solvent signals at 2.50 ppm and 7.26 ppm in DMSO-*d_6_* and CDCl_3_, respectively.

### 3.6. Determination of Apparent K_M_ Value

The kinetic parameters of FgPT1 were determined through reactions involving substrates in a buffer solution of Tris-HCl (100 mM, pH = 7.5), BaCl_2_ (1 mM), and FgPT1 (130 μg), with varying substrate concentrations. The substrates utilized were hesperitin (5.0, 2.5, 1.25, 0.625, 0.315, 0.15625, 0.078125 mM), liquiritigenin (6.25, 3.125, 1.565, 0.78125, 0.390625, 0.1953125 mM), and dihydrogenistein, alongside rac-pinocembrin and eriodictyol (1.25, 0.625, 0.3125, 0.15625, 0.078125, 0.0390625 mM). The reaction mixture was preheated to 37 °C before adding substrates to initiate the reaction. Following a 2.5 h incubation period, the reaction was quenched by adding three times the volume of ethyl acetate, and the resulting products were analyzed using HPLC. The initial enzyme activity was calculated from the linear portion of each assay, and product formation was quantified based on the ratio of the peak area of the product to that of the substrate in the HPLC analysis. Kinetic parameters were derived from a series of enzyme reactions conducted with varying substrate concentrations, utilizing GraphPad Prism 9.1 (San Diego, CA, USA) for analysis. Figure S23 illustrates the optimal fit of the Michaelis–Menten equation. All measurements were carried out in triplicate.

### 3.7. Homology Modeling and Molecular Docking Method for FgPT1

The three-dimensional structure of FgPT1 was determined through homology modeling, utilizing the substrate-bound state of the DMATS1 crystal structure (PDB: 8DAY) as a template. The protein structure of FgPT1 was constructed using the software Swiss model (version 27.0.1; SPSS Inc., Chicago, IL, USA). Subsequently, the structures of flavonoid substrates were optimized to achieve minimal energy conformations using Chem3D 21.0.0 software. The optimized ligands were then manually docked into the FgPT1 protein model, with visualization performed using PyMOL 3.7.7 software. The docking result that yielded the highest score was selected for further analysis.

### 3.8. Site-Directed Mutagenesis Method for FgPT1

The site-directed mutagenesis of FgPT1 was conducted following a previously established polymerase chain reaction (PCR) protocol [16]. The method and its improvements can be summarized as follows: Initially, primers required to synthesize mutations were designed (Table S2). The wild-type FgPT1 served as the template for PCR amplification, employing T7-For and Muts-Rev and T7-Rev and Muts-For to generate FgPT1 fragments 1 and 2, which contained the mutation sites. Subsequently, Vector-For and Vector-Rev were utilized as forward and reverse primers for PCR amplification to produce an empty fragment of pET28a. The template present in the PCR reaction product was then degraded using the DpnI enzyme. Finally, the three aforementioned fragments were ligated using the In-fusion enzyme to construct the mutant plasmid.

### 3.9. Statistical Analysis

Continuous variables are expressed as mean ± standard deviation. Data analysis utilized SPSS statistical software, applying ANOVA along with Duncan’s multiple comparisons test to assess statistical significance at *p* < 0.05.

## 4. Conclusions

This study elucidated the role of FgPT1 in mediating flavonoid prenylation, utilizing DMAPP as the prenyl donor. It demonstrated a strict substrate specificity toward six flavanones: naringenin, hesperetin, eriodictyol, liquiritigenin, rac-pinocembrin, and dihydrogenistein. The enzymatic process predominantly yielded 6-*C*-prenylated products alongside the formation of a 4′-*O*-prenylated product. Homology modeling and molecular docking studies provided insights into the binding of flavanones at the active site of FgPT1. Mutants V116I, V181I, and V194I exhibited significantly enhanced catalytic activity. These findings positioned FgPT1 as a versatile biocatalytic platform for diversifying prenylated flavanones and offered potential enzymatic tools for the targeted synthesis of bioactive prenylflavonoids.

## Figures and Tables

**Table 1:** The activities of FgPT1 towards flavonoid substrates.

Compound Name	Substrate	FgPT1	Substrate	FgPT1
Flavanones	Naringenin (**1**)	+	Hesperetin (**2**)	+
	Eriodictyol (**3**)	+	Liquiritigenin (**4**)	+
	Rac-pinocembrin (**5**)	+	Dihydrogenistein (**6**)	+
	7-Methoxyflavanone (**7**)	−	Sakuranetin (**8**)	−
	Pinostrobin (**9**)	−	2′-Hydroxyflavanone (**10**)	−
	3′-Hydroxyflavanone (**11**)	−	4′-Hydroxyflavanone (**12**)	−
	Alpinetin (**13**)	−	7-Hydroxyflavanone (**14**)	−
	6-Hydroxyflavanone (**15**)	−		
Flavanonols	Taxifolin (**16**)	−	Dihydromyricetin (**17**)	−
Chalcones	2′-Hydroxychalcone (**18**)	−	2′,4′-Dihydroxychalcone (**19**)	−
	2′,4,4′-Trihydroxychalcone (**20**)	−	4-Hydroxychalcone (**21**)	−
	4′-Hydroxychalcone (**22**)	−	2′,5′-Dihydroxychalcone (**23**)	−
	Naringenin chalcone (**24**)	−		
Flavones	Butein (**25**)	−	6-Hydroxyflavone (**26**)	−
	6,2′-Dihydroxyflavone (**27**)	−	6,3′-Dihydroxyflavone (**28**)	−
	6,4′-Dihydroxyflavone (**29**)	−	5,6-Dihydroxyflavone (**30**)	−
	6,7-Dihydroxyflavone (**31**)	−	5,6,7-Trihydroxyflavone (**32**)	−
	Flavone (**33**)	−	6-Aminoflavone (**34**)	−
	6-Methoxyflavone (**35**)	−	5-Hydroxyflavone (**36**)	−
	7-Hydroxyflavone (**37**)	−	Apigenin (**38**)	−
	Chrysin (**39**)	−	Luteolin (**40**)	−
Flavonols	3-Hydroxyflavone (**41**)	−	3,6-Dihydroxyflavone (**42**)	−
	Kaempfenrol (**43**)	−	Fisetin (**44**)	−
	Kaempferde (**45**)	−	Morin hydrate (**46**)	−
	Quercetin (**47**)	−	Myricetin (**48**)	−
Isoflavonoids	Genistein (**49**)	−	Formononetin (**50**)	−
	Daidzein (**51**)	−		
Stibenoid	Resveratrol (**53**)	−		
Prenyl flavonoid	Icaritin (**54**)	−		
Glycosilated flavonoid	Neohesperidin Dihydrochalcone (**55**)	−		
Flavan-3-ol	Catechin (**56**)	−		

Note: + and − indicated that the activity of FgPT1 was or was not detected, respectively. Bold numbers **1**–**56** indicated the substrates numbering used for FgPT1 activity assays.

**Table 2:** Kinetic parameters of FgPT1 with flavanone substrates (**1–6**).

Substrates	*k_cat_* (s^−1^)	*K_M_* (mM)	*k_cat_*/*K_M_* (s^−1^ M^−1^)
Hesperetin	0.0032 ± (0.00023)	0.94 ± (0.075)	3.40
Liquiritigenin	0.0011 ± (0.00008)	0.93 ± (0.161)	1.18
Naringenin	0.0161 ± (0.00033)	0.26 ± (0.077)	61.92
Eriodictyol	0.0014 ± (0.00015)	0.23 ± (0.026)	6.09
Dihydrogenistein	0.0007 ± (0.00008)	0.14 ± (0.026)	5.00
Pinocembrin	0.0003 ± (0.00002)	0.14 ± (0.037)	2.14

## Data Availability

All data are in the article. For questions, contact the corresponding author.

## Supplementary Materials

The following supporting information can be downloaded at: https://www.mdpi.com/article/10.3390/molecules30071558/s1, Table S1. NMR data of compounds in CDCl_3_; Table S2. Primers used for cloning; Figure S1. SDS-PAGE analysis of purified FgPT1 protein with N-His tag from *E. coli* BL21 in this study; Figure S2. Structures of substrates (**18**–**56**); Figure S3. ^1^H NMR spectrum of 6-*C*-prenylnaringenin (**1a**) in CDCl_3_ (600 MHz); Figure S4. ^13^C NMR spectrum of 6-*C*-prenyl naringenin (**1a**) in CDCl_3_ (600 MHz); Figure S5. HSQC spectrum of 6-*C*-prenyl naringenin (**1a**) in DMSO-*d_6_*; Figure S6. HMBC spectrum of 6-*C*-prenyl naringenin (**1a**) in DMSO-*d_6_*; Figure S7. ^1^H NMR spectrum of 6-*C*-prenyl hesperetin (**2a**) in CDCl_3_ (600 MHz); Figure S8. ^13^C NMR spectrum of 6-*C*-prenyl hesperetin (**2a**) in CDCl_3_ (600 MHz); Figure S9. HSQC spectrum of 6-*C*-prenyl hesperetin (**2a**) in CDCl_3_; Figure S10. HMBC spectrum of 6-*C*-prenyl hesperetin (**2a**) in CDCl_3_; Figure S11. ^1^H NMR spectrum of 6-*C*-prenyl eriodictyol (**3a**) in CDCl_3_ (600 MHz); Figure S12. ^13^C NMR spectrum of 6-*C*-prenyl eriodictyol (**3a**) in CDCl_3_ (600 MHz); Figure S13. ^1^H NMR spectrum of 6-*C*-prenyl dihydrogenistein (**6a**) in CDCl_3_ (600 MHz); Figure S14. ^13^C NMR spectrum of 6-*C*-prenyl dihydrogenistein (**6a**) in CDCl_3_ (600 MHz); Figure S15. HSQC spectrum of 6-*C*-prenyl dihydrogenistein (**6a**) in CDCl_3_; Figure S16. HMBC spectrum of 6-*C*-prenyl dihydrogenistein (**6a**) in CDCl_3_; Figure S17. ^1^H NMR spectrum of 6-*C*-prenyl liquiritigenin (**4a1**) in CDCl_3_ (600 MHz); Figure S18. ^13^C NMR spectrum of 6-*C*-prenyl liquiritigenin (**4a1**) in CDCl_3_ (600 MHz); Figure S19. ^1^H NMR spectrum of 4’-*O*-prenyl liquiritigenin (**4a2**) in CDCl_3_ (600 MHz); Figure S20. ^13^C NMR spectrum of 4’-*O*-prenyl liquiritigenin (**4a2**) in CDCl_3_ (600 MHz); Figure S21. HSQC spectrum of 4’-*O*-prenyl liquiritigenin (**4a2**) in CDCl_3_; Figure S22. HMBC spectrum of 4’-*O*-prenyl liquiritigenin (**4a2**) in CDCl_3_; Figure S23. Kinetic parameters of the FgPT1 reaction of substrates (**1**–**6**) in the presence of DMAPP; Figure S24. The activity of FgPT1 (wild type and 7 mutants) on substrates (**1**–**6**); Figure S25. The docking model of FgPT1 (325 site). The graphical abstract.
