# Supplementary material for: Prenylation of Flavanones by an Aromatic Prenyltransferase from Fusarium globosum
Source: Molecules. 2025 Mar 31;30(7):1558. doi: 10.3390/molecules30071558 (PMC11990136; doi:10.3390/molecules30071558)
Supplement: Supplementary file 1 [file molecules-30-01558-s001.zip › molecules-3539534-supplementary.pdf]

Table S1. NMR data of compounds in $CDCl_3$.

Table S2. Primers used for cloning.

Figure S1. SDS-PAGE analysis of purified FgPT1 protein with N-His tag from *E. coli* BL21 in this study. SF, soluble protein fraction after centrifugation; WF, washing fraction; EF, elution fraction; M, marker.

Figure S2. Structures of substrates (**18-56**).

Figure S3. ^{1}H NMR spectrum of 6-*C*-prenylnaringenin (**1a**) in $CDCl_3$ (600 MHz).

Figure S4. ^{13}C NMR spectrum of 6-*C*-prenyl naringenin (**1a**) in $CDCl_3$ (600 MHz).

Figure S5. HSQC spectrum of 6-*C*-prenyl naringenin (**1a**) in DMSO-d_6.

Figure S6. HMBC spectrum of 6-*C*-prenyl naringenin (**1a**) in DMSO-d_6.

Figure S7. ^{1}H NMR spectrum of 6-*C*-prenyl hesperetin (**2a**) in $CDCl_3$ (600 MHz).

Figure S8. ^{13}C NMR spectrum of 6-*C*-prenyl hesperetin (**2a**) in $CDCl_3$ (600 MHz).

Figure S9. HSQC spectrum of 6-*C*-prenyl hesperetin (**2a**) in $CDCl_3$.

Figure S10. HMBC spectrum of 6-*C*-prenyl hesperetin (**2a**) in $CDCl_3$.

Figure S11. ^{1}H NMR spectrum of 6-*C*-prenyl eriodictyol (**3a**) in $CDCl_3$ (600 MHz).

Figure S12. ^{13}C NMR spectrum of 6-*C*-prenyl eriodictyol (**3a**) in $CDCl_3$ (600 MHz).

Figure S13. ^{1}H NMR spectrum of 6-*C*-prenyl dihydrogenistein (**6a**) in $CDCl_3$ (600 MHz).

Figure S14. ^{13}C NMR spectrum of 6-*C*-prenyl dihydrogenistein (**6a**) in $CDCl_3$ (600 MHz).

Figure S15. HSQC spectrum of 6-*C*-prenyl dihydrogenistein (**6a**) in $CDCl_3$.

Figure S16. HMBC spectrum of 6-*C*-prenyl dihydrogenistein (**6a**) in $CDCl_3$.

Figure S17. ^{1}H NMR spectrum of 6-*C*-prenyl liquiritigenin (**4a1**) in $CDCl_3$ (600 MHz).

Figure S18. ^{13}C NMR spectrum of 6-*C*-prenyl liquiritigenin (**4a1**) in $CDCl_3$ (600 MHz).

Figure S19. ^{1}H NMR spectrum of 4′-*O*-prenyl liquiritigenin (**4a2**) in $CDCl_3$ (600 MHz).

Figure S20. ^{13}C NMR spectrum of 4′-*O*-prenyl liquiritigenin (**4a2**) in $CDCl_3$ (600 MHz).

Figure S21. HSQC spectrum of 4′-*O*-prenyl liquiritigenin (**4a2**) in $CDCl_3$.

Figure S22. HMBC spectrum of 4′-*O*-prenyl liquiritigenin (**4a2**) in $CDCl_3$.

Figure S23. Kinetic parameters of the FgPT1 reaction of substrates (**1-6**) in the presence of DMAPP. (A) naringenin (**1**). (B) hesperitin (**2**), (C) eriodictyol (**3**), (D) liquiritigenin (**4**), (E) rac-pinocembrin (**5**), (F) dihydrogenistein (**6**).

Figure 24. The activity of FgPT1 (wild type and 7 mutants) on substrates (**1-6**).

Figure S25. The docking model of FgPT1 (325 site). (A) naringenin, F326 and A325. (B) naringenin, F326 and V325.

Table S1. NMR data of compounds in $CDCl_3$.

6-Prenyl Naringenin			6-Prenyl Hesperetin			6-Prenyl Eriodictyol		
position	δ_H, multiplicity (*J* in Hz)	δ_C	position	δ_H, multiplicity (J in Hz)	δ_C	position	δ_H, multiplicity (J in Hz)	δ_C
2	5.38 (dd, *J* = 12.8, 3.1 Hz)	78.74	2	5.29 (dd, *J* = 12.9, 3.0 Hz)	78.94	2	5.32 (dd, *J* = 12.4, 3.1 Hz)	78.75
3	3.21 (dd, *J* = 17.1, 12.8 Hz)	42.51	3	3.06 (dd, *J* = 17.1, 12.9 Hz)	43.40	3	3.14 (dd, *J* = 17.1, 12.4 Hz)	42.60
	2.65 (dd, *J* = 17.1, 3.1 Hz)			2.77 (dd, *J* = 17.1, 3.0 Hz)			2.65 (dd, *J* = 17.1, 3.1 Hz)	
4		196.58	4		196.21	4		196.35
4a		101.77	4a		103.04	4a		101.33
5		160.97	5		161.35	5		160.91
6		108.03	6		107.04	6		107.97
7		165.49	7		163.91	7		163.37
8	5.92, s	94.97	8	5.99, s	95.68	8	5.92, s	160.95
8a		160.97	8a		161.16	8a		94.93
1′		129.52	1′		131.77	1′		130.09
2′	7.30 (d, *J* = 8.6 Hz)	128.75	2′	6.92 (dd, *J* = 8.3, 2.1 Hz)	118.28	2′	6.73, s	114.73
3′	6.78 (d, *J* = 8.6 Hz)	115.60	3′	6.87 (d, *J* = 8.3 Hz)	110.75	3′		145.64
4′		158.14	4′		147.06	4′		146.11
5′	6.78 (d, *J* = 8.6 Hz)	115.60	5′		146.00	5′	6.73 (d, *J* = 1.8 Hz)	118.32
6′	7.30 (d, *J* = 8.6 Hz)	128.75	6′	7.04 (d, *J* = 2.1 Hz)	112.78	6′	6.86 (d, *J* = 1.8 Hz)	120.84
1′′	3.10 (d, *J* = 7.1 Hz)	21.11	1′′	3.35 (d, *J* = 7.2 Hz)	21.26	1′′	3.10 (d, *J* = 7.4 Hz)	21.10
2′′	5.15 - 5.09 (brt, *J* = 7.1 Hz)	123.25	2′′	5.27 - 5.22 (brt, *J* = 7.2 Hz)	121.58	2′′	5.15 - 5.09 (brt, *J* = 7.4 Hz)	123.23
3′′		130.58	3′′		135.73	3′′		134.71
4′′	1.69, s	18.13	4′′	1.82, s	18.03	4′′	1.69, s	18.13
5′′	1.60, s	25.96	5′′	1.76, s	25.99	5′′	1.61, s	25.95
			1′′′	3.91, s	56.18			

Table S1. (continued).

6-Prenyl Dihydrogenistein			6-Prenyl Liquiritigenin			4′-Prenyl Liquiritigenin		
position	δ_H, multiplicity (J in Hz)	δ_C	position	δ_H, multiplicity (J in Hz)	δ_C	position	δ_H, multiplicity (J in Hz)	δ_C
2	4.55 (dd, *J* = 11.2, 5.1 Hz)	71.30	2	5.39 (dd, *J* = 12.9, 2.9 Hz)	79.41	2	5.40 (dd, *J* = 13.4, 2.8 Hz)	79.70
	4.50 (dd, *J* = 11.2, 8.5 Hz)							
3	3.88 (dd, *J* = 8.5, 5.1 Hz)	50.52	3	2.60 (dd, *J* = 16.8, 2.9 Hz)	43.69	3	3.05 (dd, *J* = 16.8, 13.4 Hz)	44.10
				3.07 (dd, *J* = 16.8, 12.9 Hz)			2.80 (dd, *J* = 16.8, 2.8 Hz)	
4		196.75	4		190.62	4		191.11
4a		102.85	4a		113.57	4a		115.03
5		161.71	5	7.44, s	127.24	5	7.85 (dd, *J* = 8.7, 1.3 Hz)	129.39
6		106.96	6		122.72	6	6.54 (dd, *J* = 8.7, 1.3 Hz)	110.56
7		163.38	7		162.90	7		163.66
8	5.97, s	95.25	8	6.37, s	102.56	8	6.45, s	103.42
8a		161.05	8a		161.86	8a		162.88
1′		127.12	1′		129.91	1′		130.63
2′	7.15 (d, *J* = 8.0 Hz)	129.87	2′	7.31 (d, *J* = 8.6 Hz)	128.68	2′	7.38 (d, *J* = 7.5 Hz)	127.76
3′	6.81 (d, *J* = 8.0 Hz)	115.86	3′	6.78 (d, *J* = 8.5 Hz)	115.60	3′	6.96 (d, *J* = 7.5 Hz)	114.90
4′		155.31	4′		158.06	4′		159.27
5′	6.81 (d, *J* = 8.0 Hz)	115.86	5′	6.78 (d, *J* = 8.5 Hz)	115.60	5′	6.96 (d, *J* = 7.5 Hz)	114.90
6′	7.15 (d, *J* = 8.0 Hz)	129.87	6′	7.31 (d, *J* = 8.6 Hz)	128.68	6′	7.38 (d, *J* = 7.5 Hz)	127.76
1′′	3.34 (d, *J* = 7.2 Hz)	21.18	1′′	3.16 (d, *J* = 7.4 Hz)	27.62	1′′	4.53 (d, *J* = 6.8 Hz)	64.87
2′′	5.27 - 5.22 (brt, *J* = 7.2 Hz)	121.36	2′′	5.29 - 5.21 (brt, *J* = 7.4 Hz)	123.08	2′′	5.52 - 5.47 (brt, *J* = 6.8 Hz)	119.41
3′′		135.81	3′′		132.34	3′′		138.59
4′′	1.81, s	17.92	4′′	1.70, s	18.07	4′′	1.75, s	18.25
5′′	1.76, s	25.87	5′′	1.65, s	26.04	5′′	1.80, s	25.88

Table S2. Primers used for cloning.

Primer name	Primer sequence (5′→3′)
T7 - F	TAATACGACTCACTATAGGG
T7 - R	GCTAGTTATTGCTCAGCGGT
Pet28a - F	TGAGCAATAACTAGCATAACCCCTTGGG
Pet28a - R	TAGTGAGTCGTATTAATTTCGCGGGATC
V1116I - F	ACGCCATTGAACTCGTTAGTCCCTTAACC
V116I - R	CGAGTTCAATGGCGTATCTAATGTCAGGC
T123A - F	GTCCCTTAGCCGGAAGCAAGCAAGATC
T123A - R	TTCCGGCAAGGGACTAACGAGTTCAACG
V181I - F	CAACTATTTTTGCGGCCTTGGAAATGC
V181I - R	GCCGCAAAAATAGTTGAACCCTTTGTCGAAATTC
G190D - F	CTCCACGATCACTTGTCCGTCAAAGTG
G190D - R	CAAGTGATCGTGGAGCATTTCCAAGG
V194I - F	CTTGTCCATCAAGTGTATTACATACCACTCG
V194I - R	ACTTTGATGGACAAGTGACCGTGGAG
Y198F - F	GTATTTCATACCACTCGAAACACCGGATTTC
Y198F - R	AGTGGTATGAAATACACTTTGACGGACAAG
A325V - F	GGGCGGTATTTAACTTTGACGTCGC
A325V - R	AGTTAAATACCGCCCCGGAAGTCAGATG

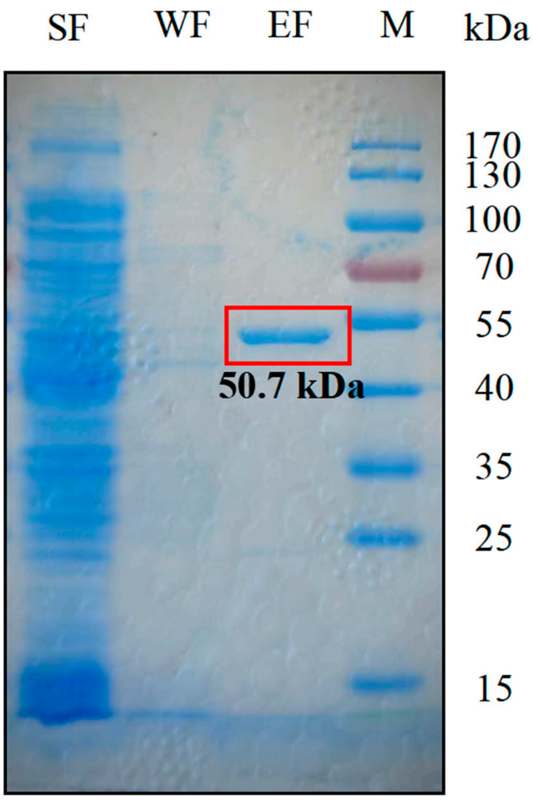

Figure S1. SDS-PAGE analysis of purified FgPT1 protein with N-His tag from *E. coli* BL21 in this study. SF, soluble protein fraction after centrifugation; WF, washing fraction; EF, elution fraction; M, marker.

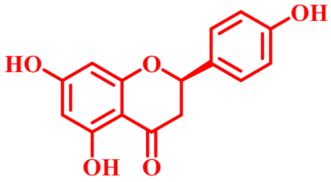

Naringenin (**1**)

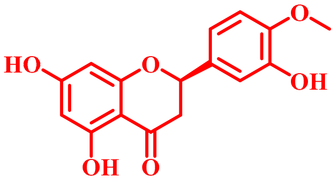

Hesperetin (**2**)

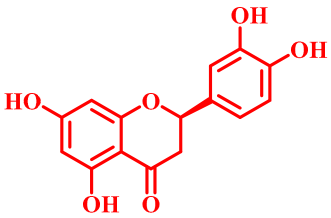

Eriodictyol (**3**)

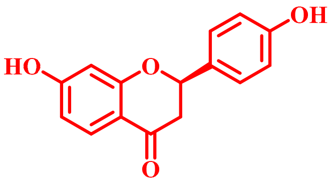

Liquiritigenin (**4**)

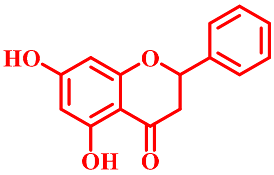

Rac-pinocembrin (**5**)

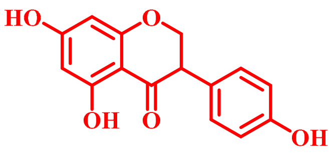

Dihydrogenistein (**6**)

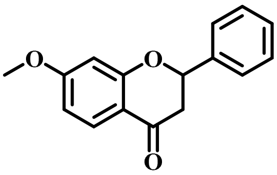

7-Methoxyflavanone (**7**)

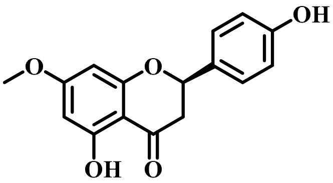

Sakuranetin (**8**)

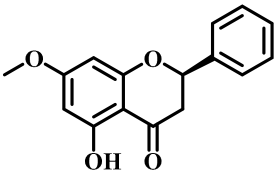

Pinostrobin (**9**)

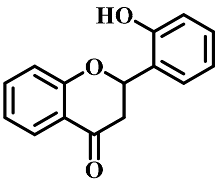

2'-Hydroxyflavanone (**10**)

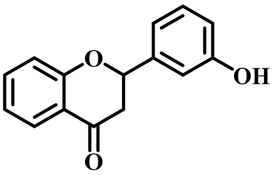

3'-Hydroxyflavanone (**11**)

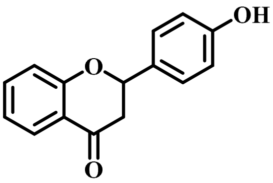

4'-Hydroxyflavanone (**12**)

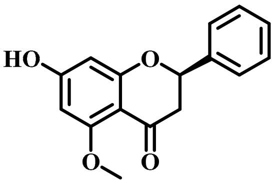

Alpinetin (**13**)

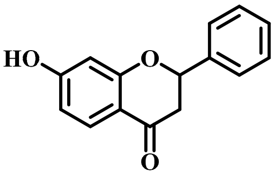

7-Hydroxyflavanone (**14**)

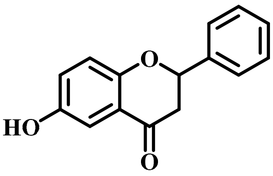

6-Hydroxyflavanone (**15**)

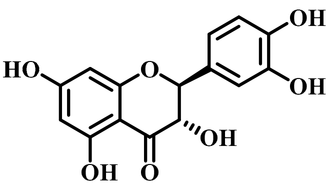

Taxifolin (**16**)

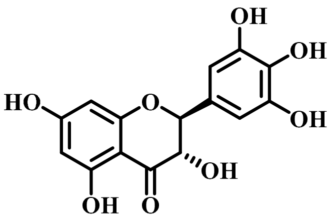

Dihydromyricetin (**17**)

Figure S2. Structures of substrates (**18-56**).

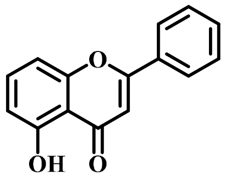

5-Hydroxyflavone (**36**)

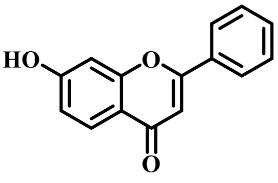

7-Hydroxyflavone (**37**)

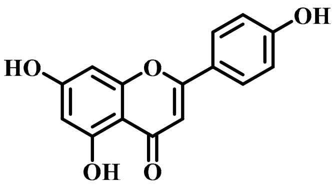

Apigenin (**38**)

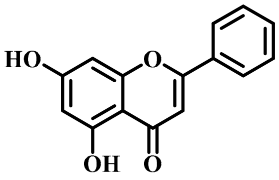

Chrysin (**39**)

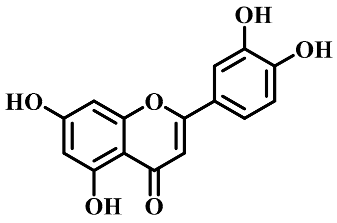

luteolin (**40**)

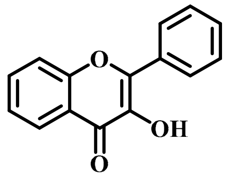

3-Hydroxyflavone (**41**)

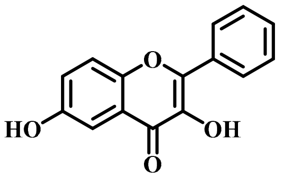

3,6-Dihydroxyflavone (**42**)

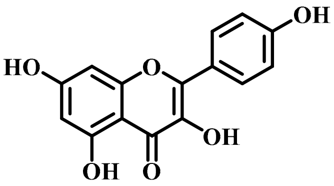

Kaempferol (**43**)

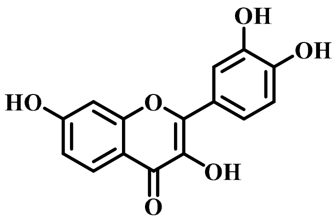

Fisetin (**44**)

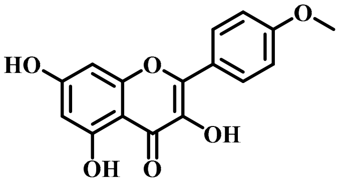

Kaempferide (**45**)

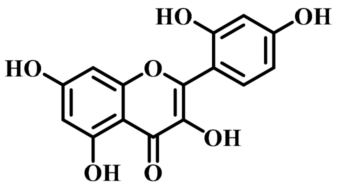

Morin hydrate (**46**)

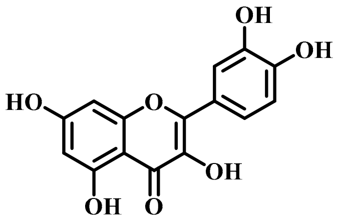

Quercetin (**47**)

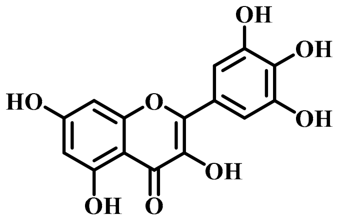

Myricetin (**48**)

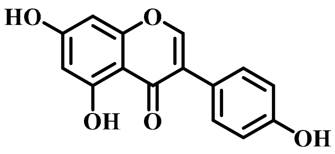

Genistein (**49**)

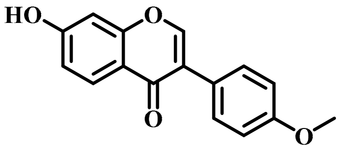

Formononetin (**50**)

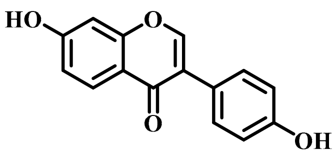

Daidzein (**51**)

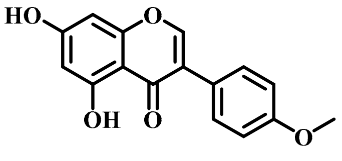

Biochanin A (**52**)

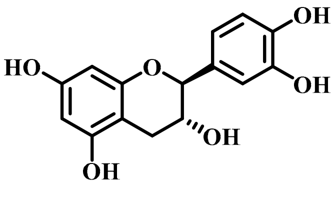

Catechin (**53**)

Figure S2. (continued).

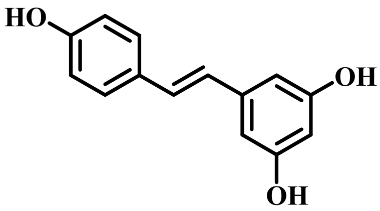

Resveratrol (**54**)

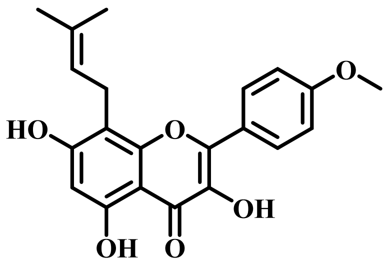

Icaritin (**55**)

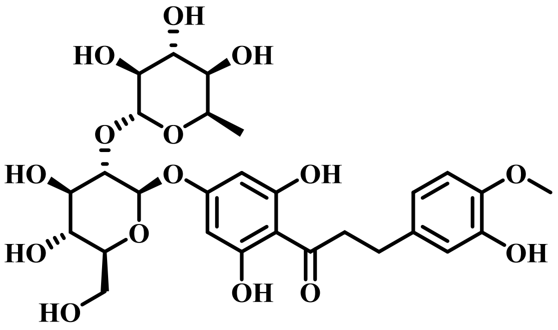

Neohesperidin Dihydrochalcone (**56**)

Figure S2. (continued).

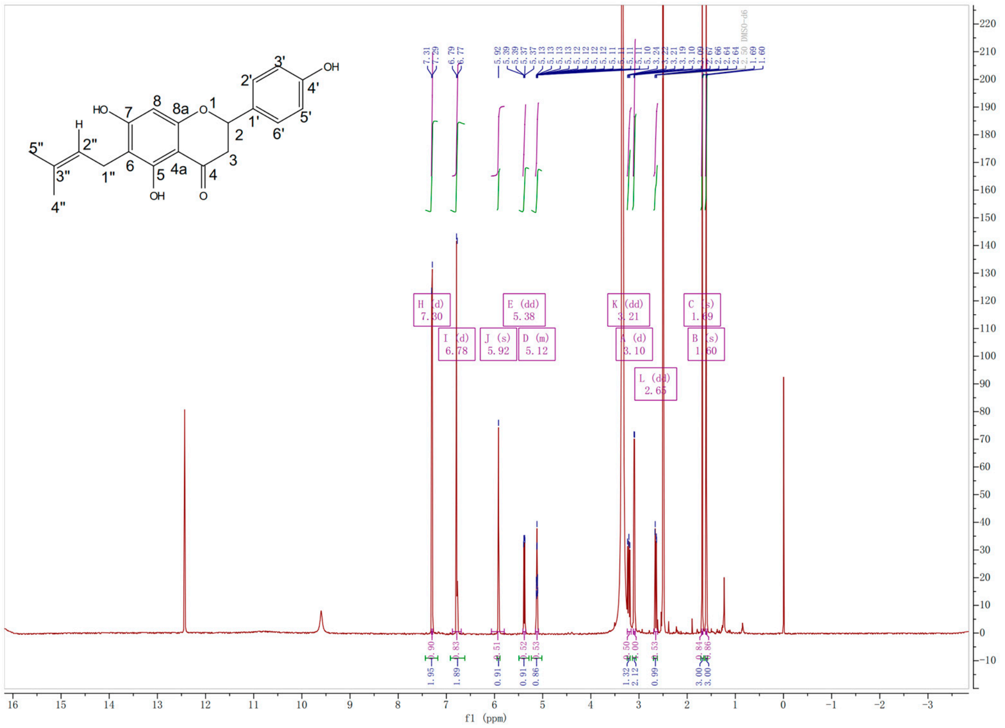

Figure S3. ^{1}H NMR spectrum of 6-*C*-prenylnaringenin (**1a**) in $CDCl_3$ (600 MHz).

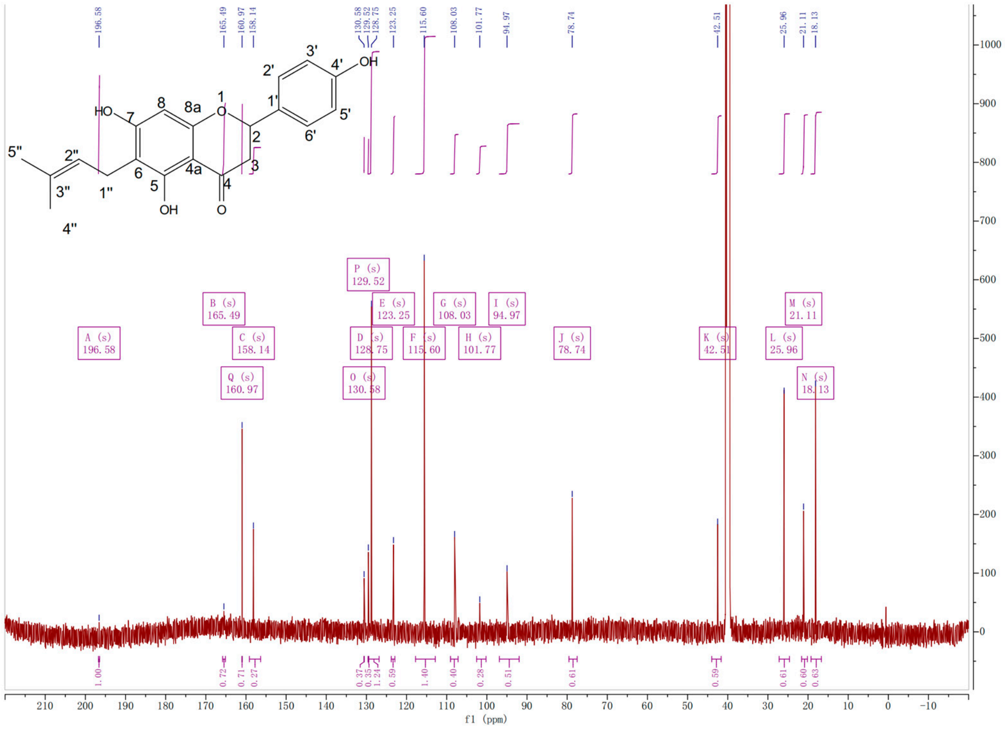

Figure S4. ^{13}C NMR spectrum of 6-*C*-prenyl naringenin (**1a**) in $CDCl_3$ (600 MHz).

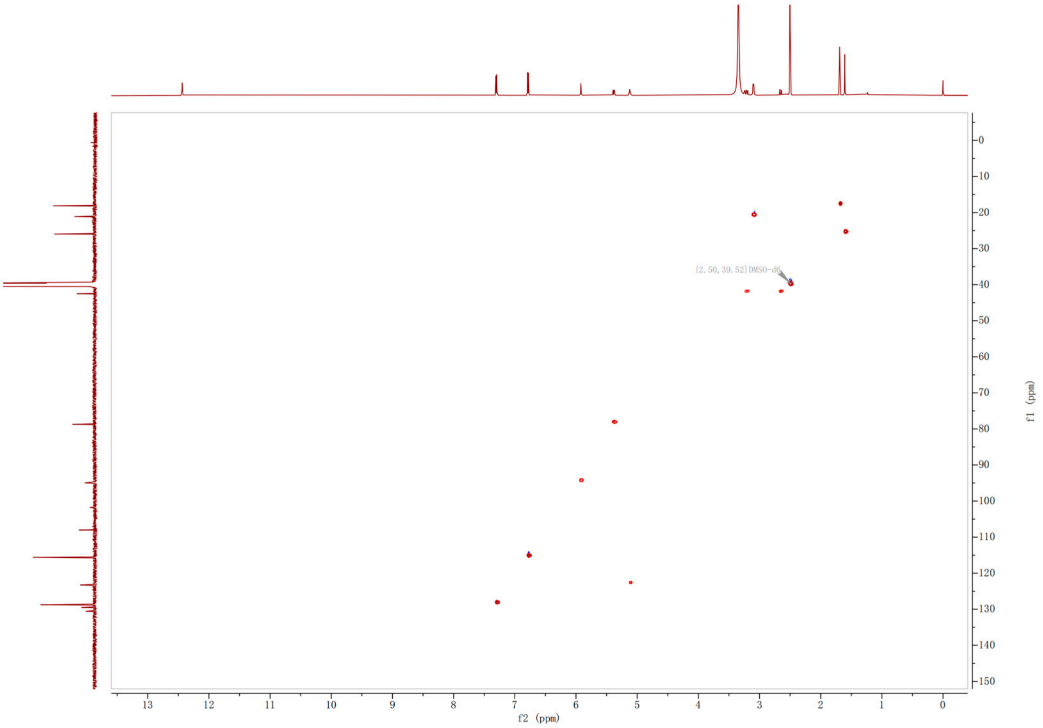

Figure S5. HSQC spectrum of 6-*C*-prenyl naringenin (**1a**) in DMSO-*d*$_6$.

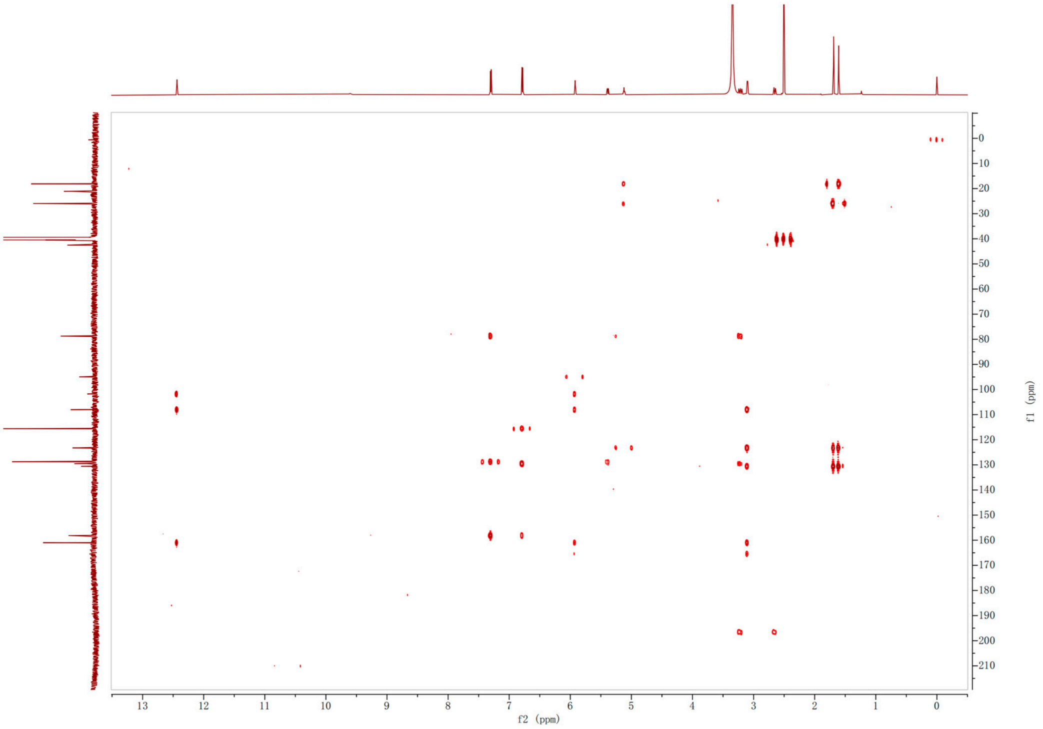

Figure S6. HMBC spectrum of 6-*C*-prenyl naringenin (**1a**) in DMSO-*d*$_6$.

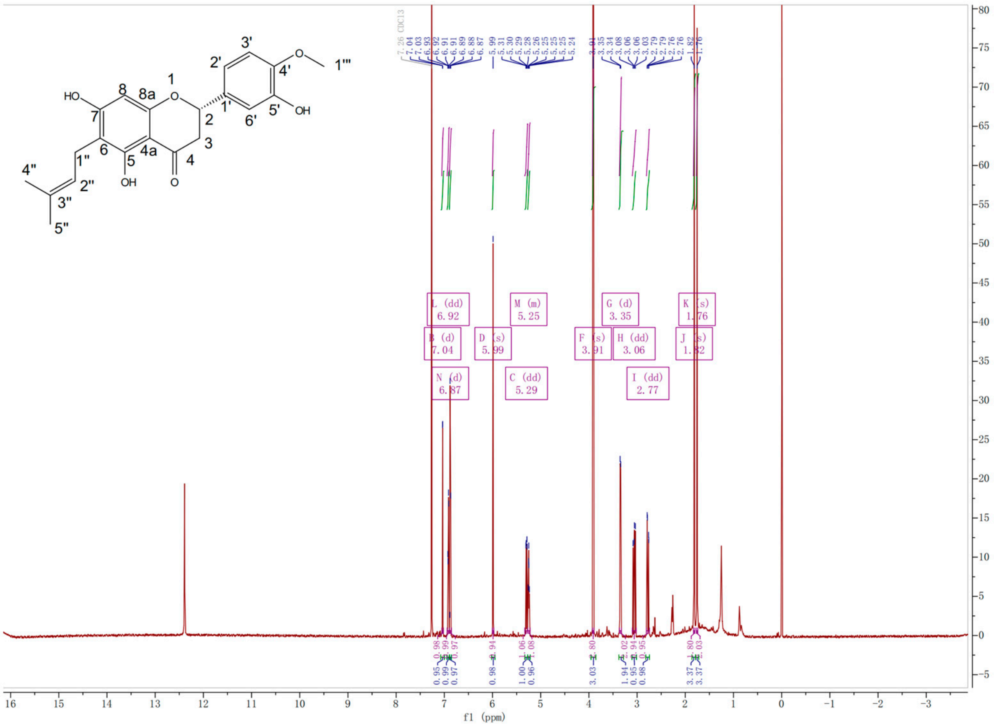

Figure S7. ^{1}H NMR spectrum of 6-*C*-prenyl hesperetin (**2a**) in $CDCl_3$ (600 MHz).

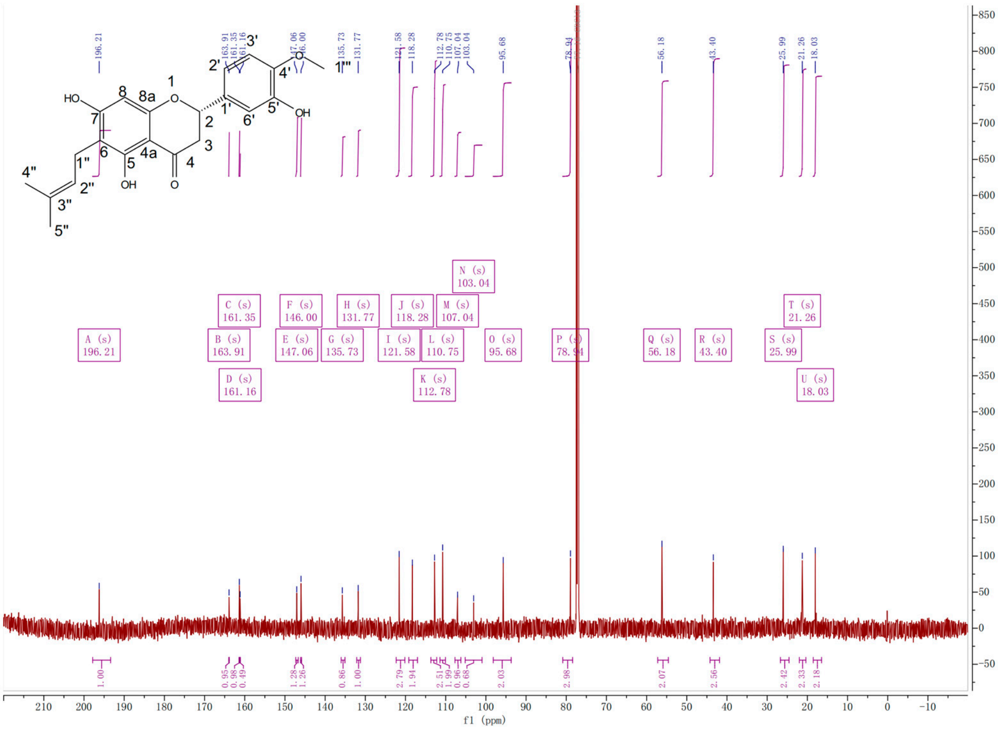

Figure S8. ^{13}C NMR spectrum of 6-*C*-prenyl hesperetin (**2a**) in $CDCl_3$ (600 MHz).

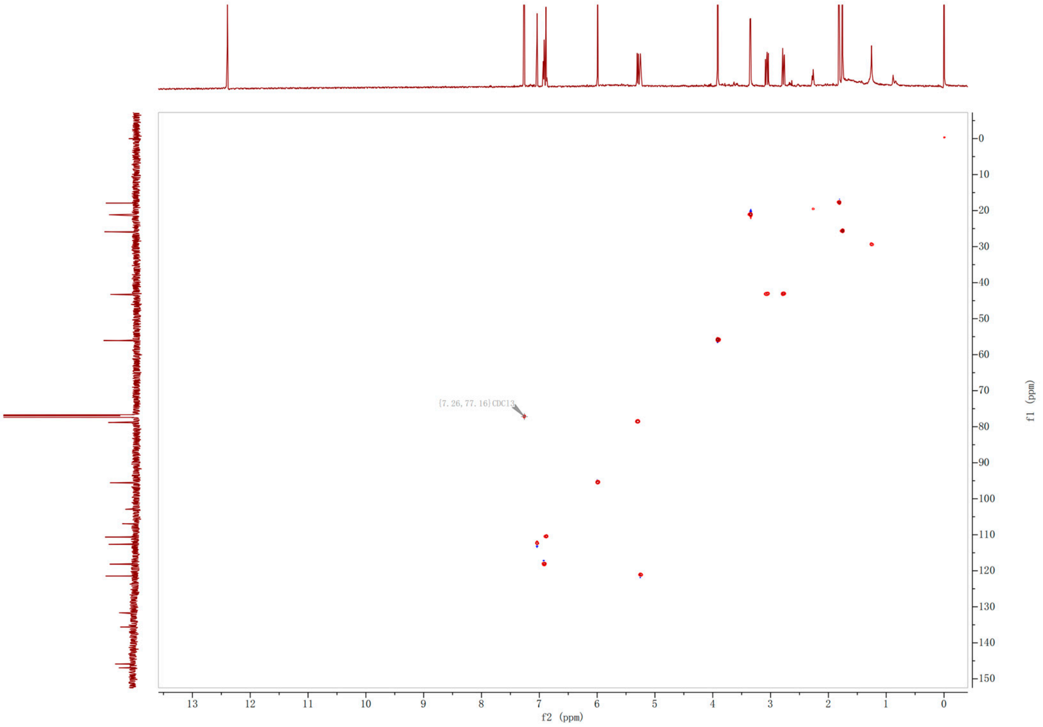

Figure S9. HSQC spectrum of 6-*C*-prenyl hesperetin (**2a**) in $CDCl_3$.

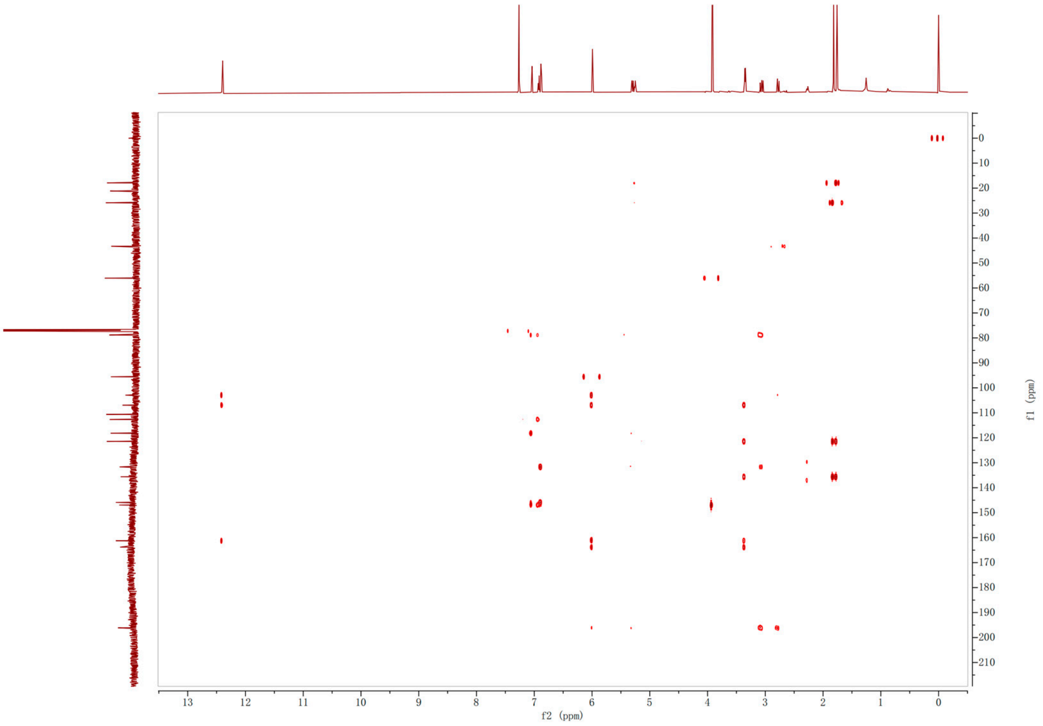

Figure S10. HMBC spectrum of 6-*C*-prenyl hesperetin (**2a**) in $CDCl_3$.

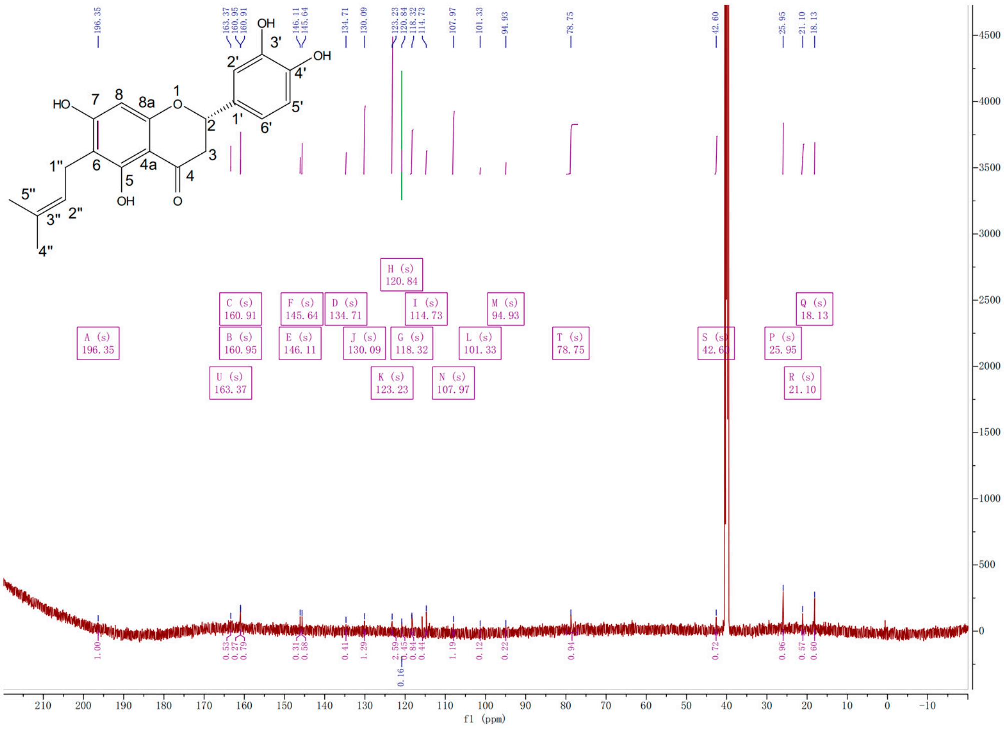

Figure S11. ^{1}H NMR spectrum of 6-*C*-prenyl eriodictyol (**3a**) in CDCl3 (600 MHz).

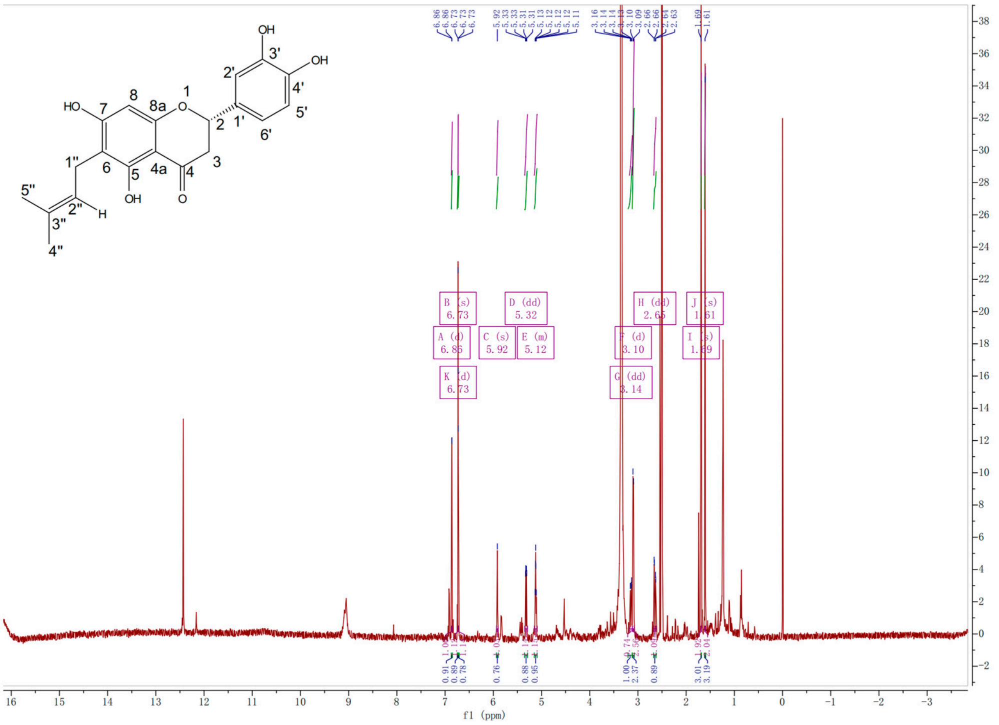

Figure S12. ^{13}C NMR spectrum of 6-*C*-prenyl eriodictyol (**3a**) in CDCl3 (600 MHz).

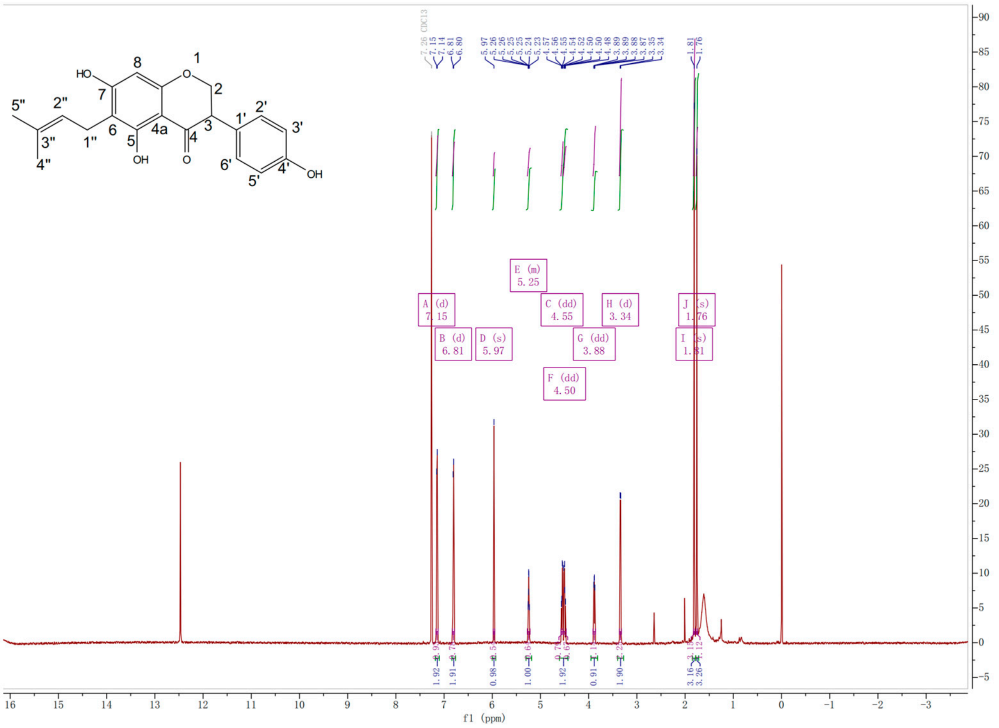

Figure S13. 1H NMR spectrum of 6-C-prenyl dihydrogenistein (6a) in CDCl3 (600 MHz).

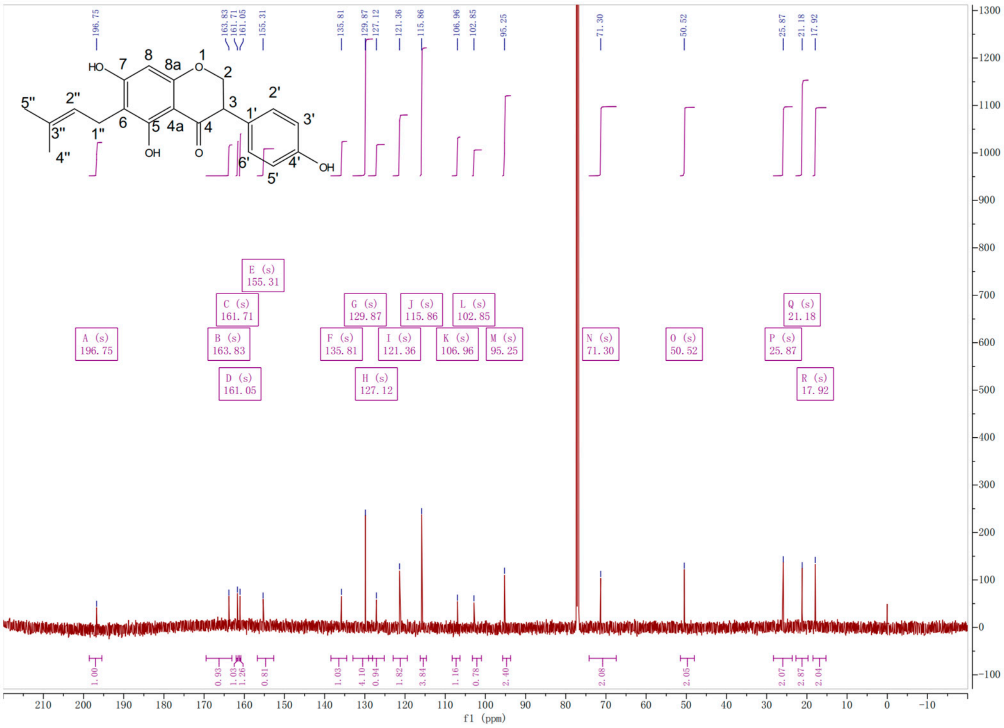

Figure S14. ^{13}C NMR spectrum of 6-*C*-prenyl dihydrogenistein (**6a**) in $CDCl_3$ (600 MHz).

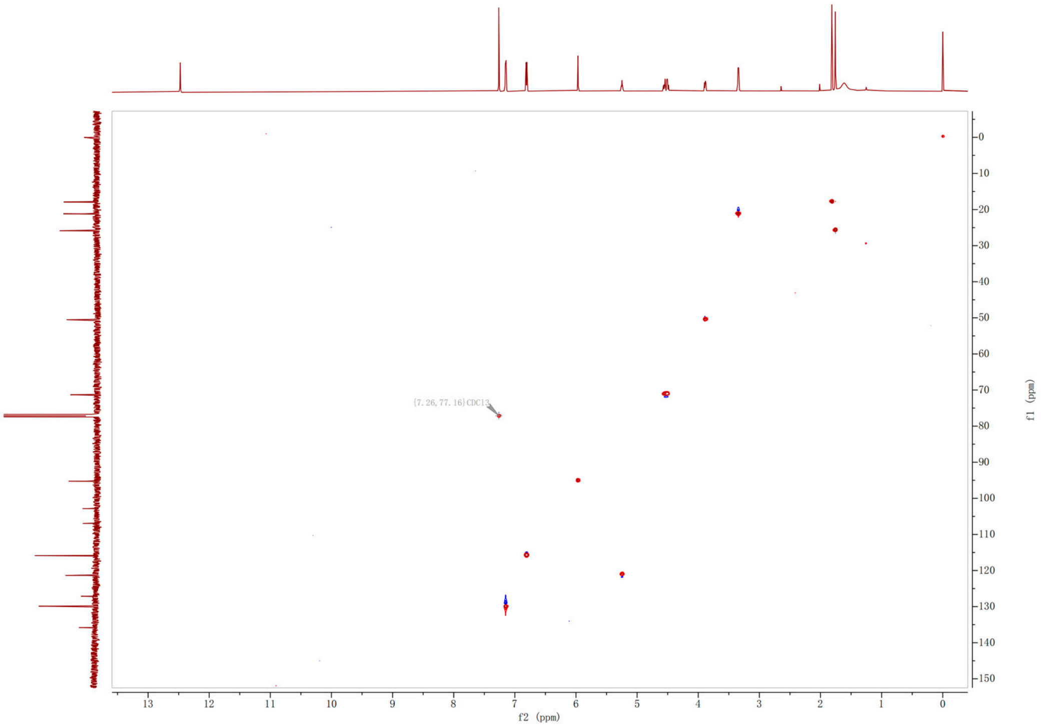

Figure S15. HSQC spectrum of 6-*C*-prenyl dihydrogenistein (**6a**) in $CDCl_3$.

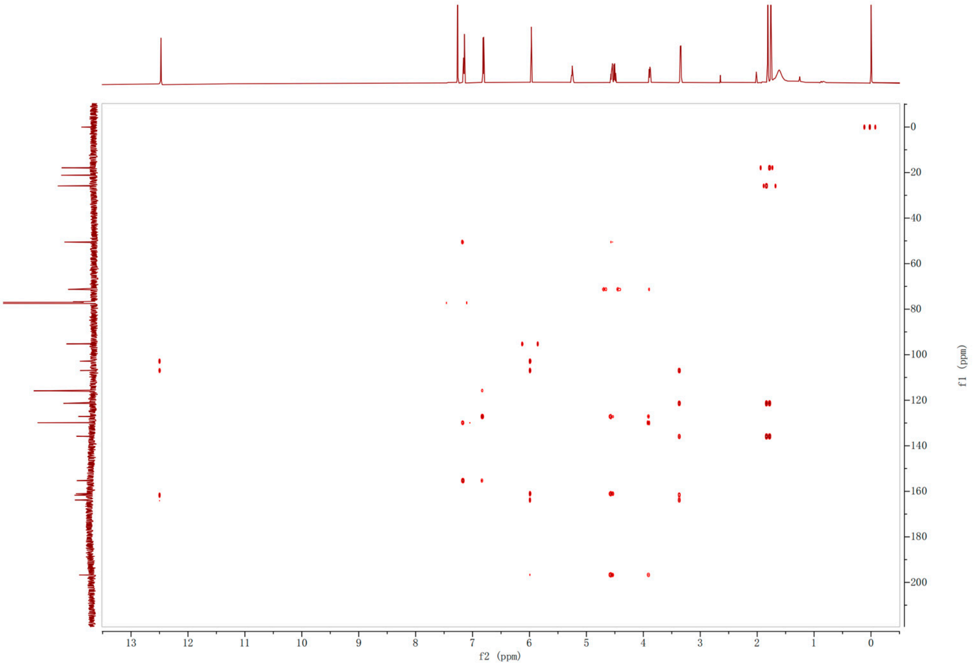

Figure S16. HMBC spectrum of 6-*C*-prenyl dihydrogenistein (**6a**) in $CDCl_3$.

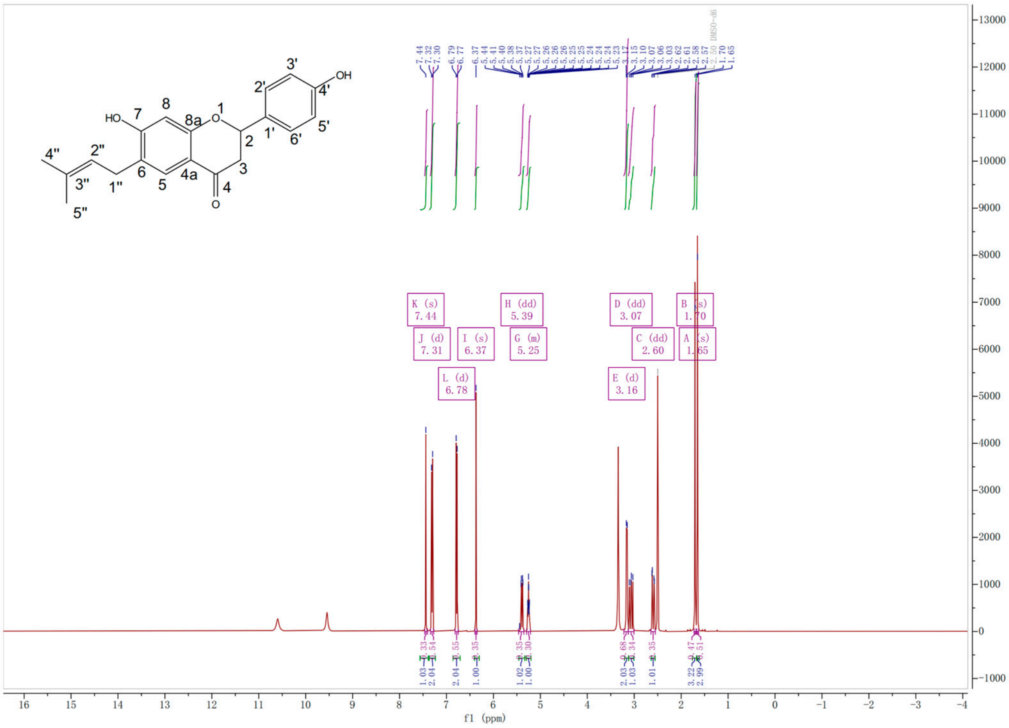

Figure S17. ^{1}H NMR spectrum of 6-*C*-prenyl liquiritigenin (**4a1**) in $CDCl_3$ (600 MHz).

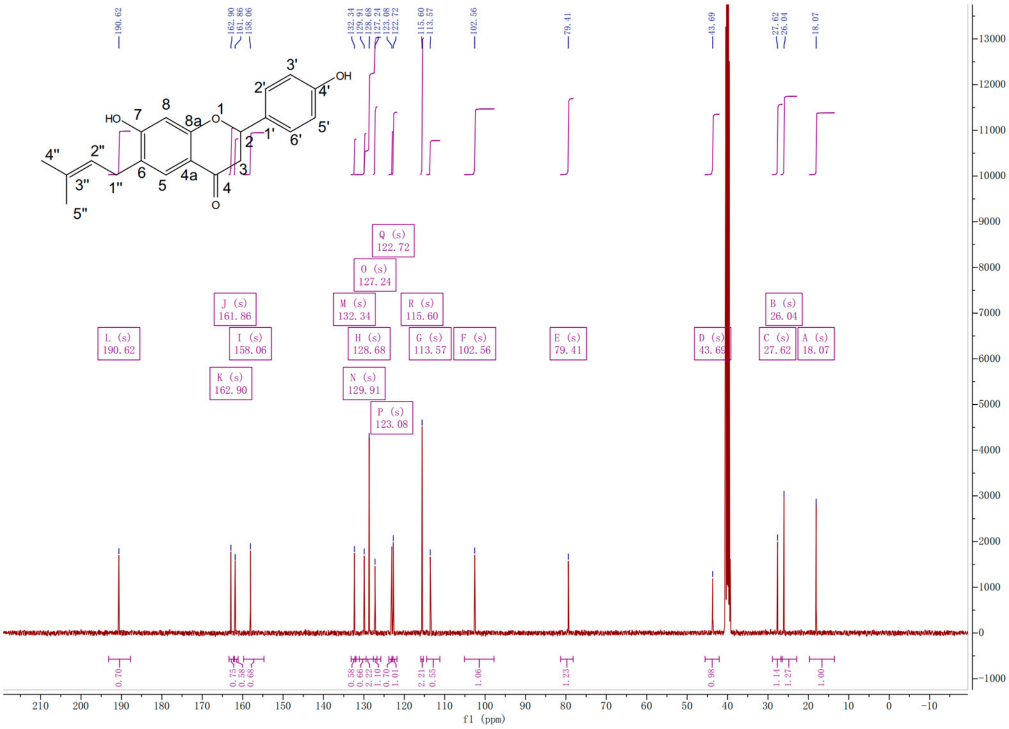

Figure S18. ^{13}C NMR spectrum of 6-*C*-prenyl liquiritigenin (**4a1**) in $CDCl_3$ (600 MHz).

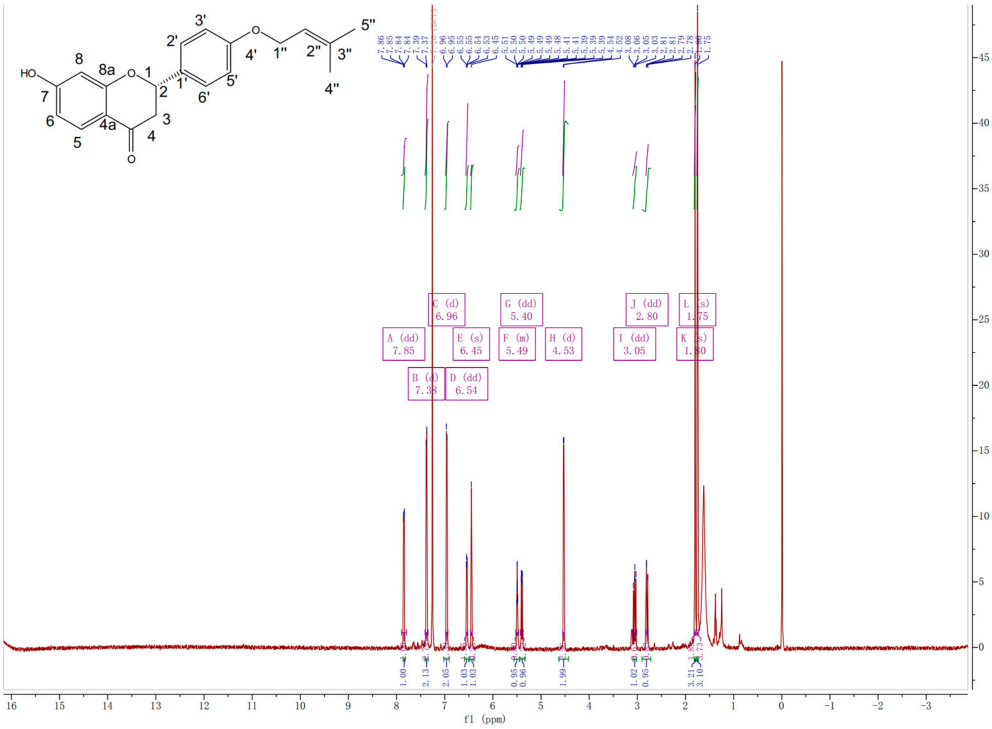

Figure S19. ^{1}H NMR spectrum of 4′-*O*-prenyl liquiritigenin (**4a2**) in $CDCl_3$ (600 MHz).

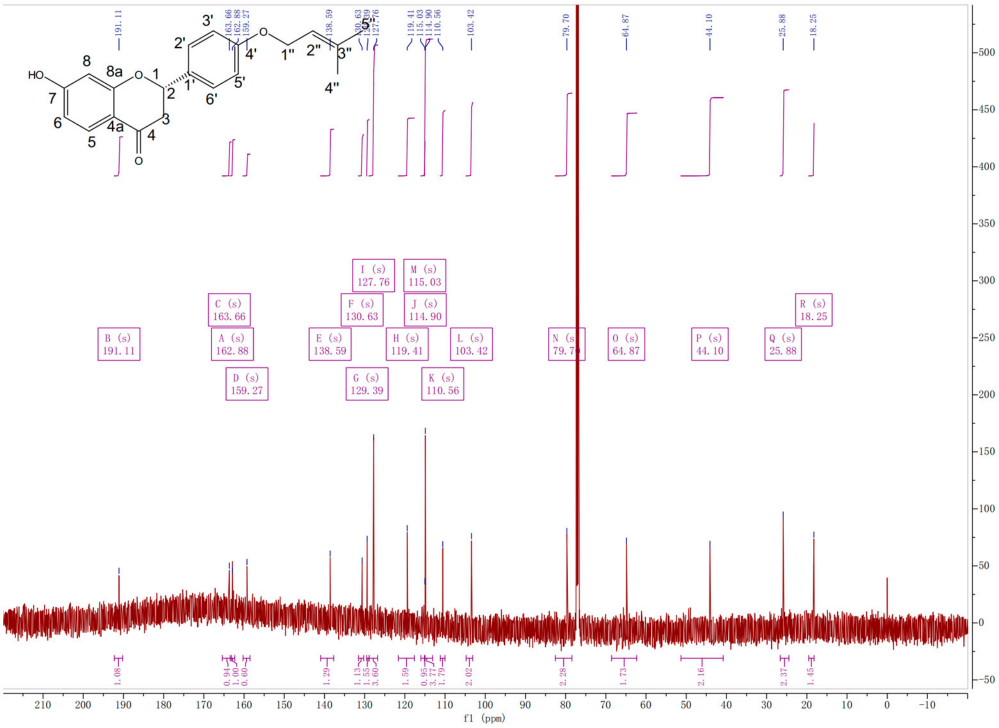

Figure S20. ^{13}C NMR spectrum of 4′-*O*-prenyl liquiritigenin (**4a2**) in $CDCl_3$ (600 MHz).

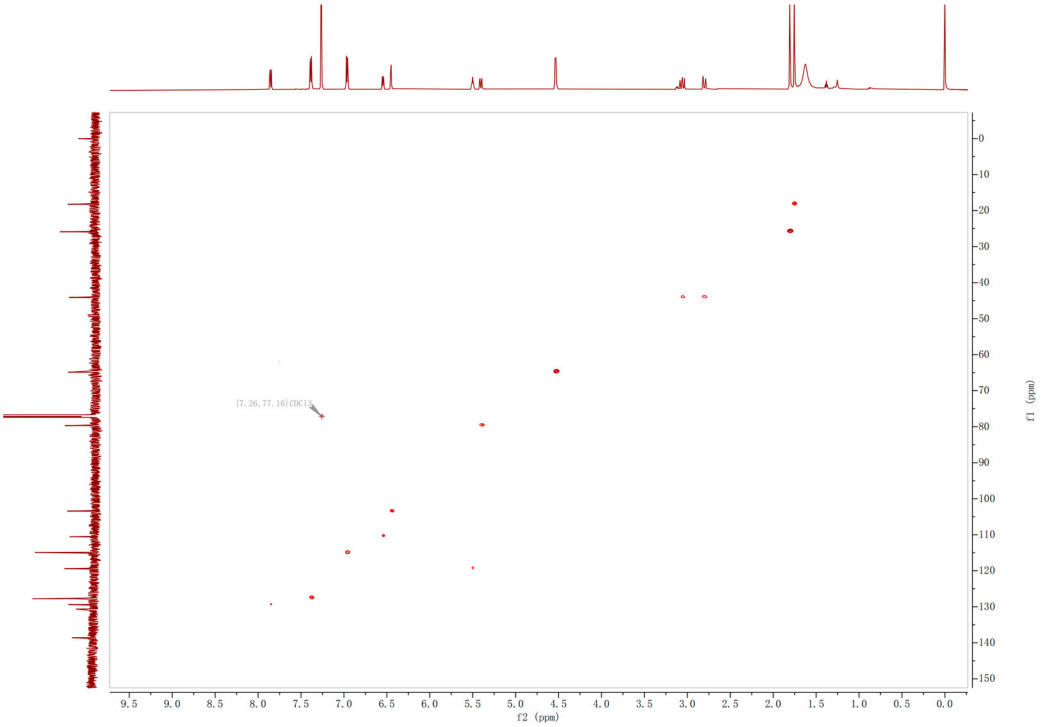

Figure S21. HSQC spectrum of 6-*O*-prenyl liquiritigenin (**4a2**) in $CDCl_3$.

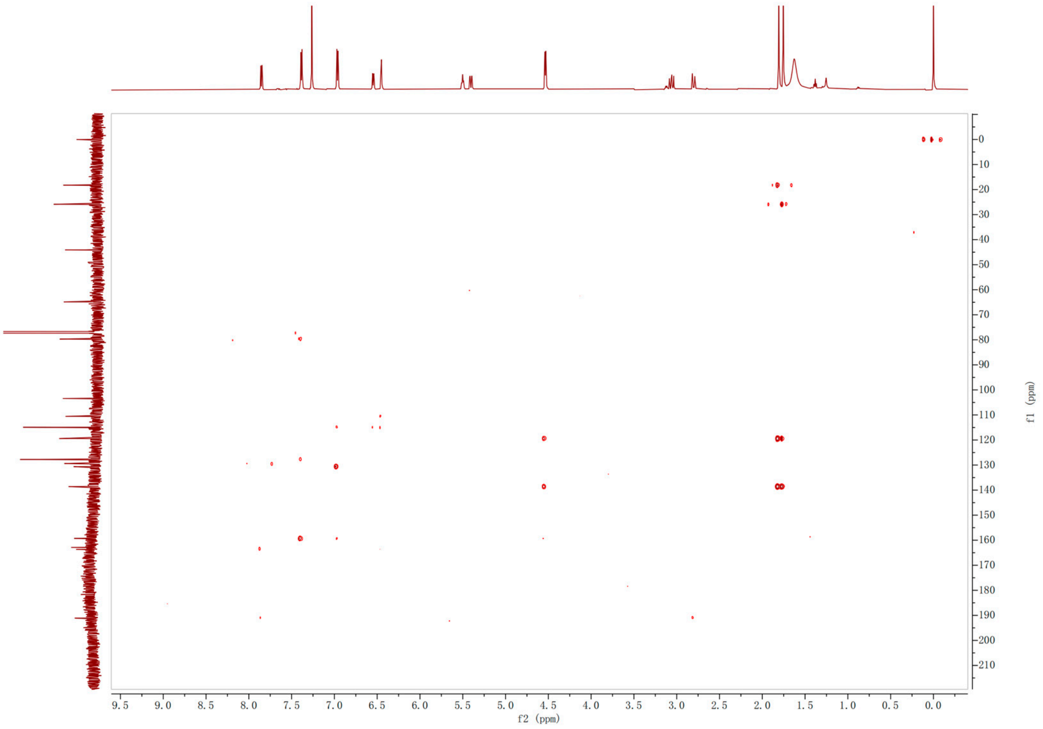

Figure S22. HMBC spectrum of 4′-*O*-prenyl liquiritigenin (**4a2**) in $CDCl_3$.

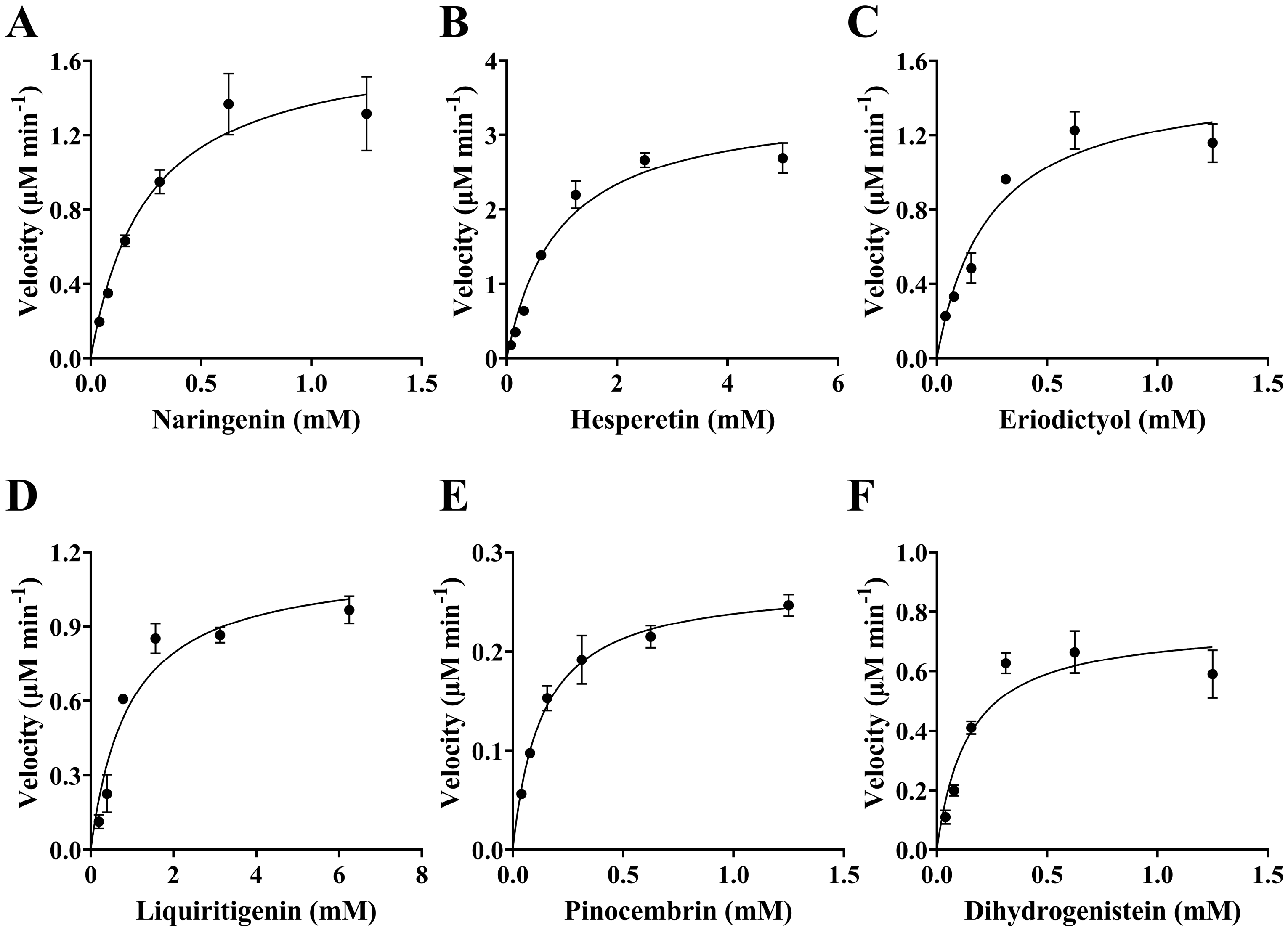

Figure S23. Kinetic parameters of the FgPT1 reaction of substrates (**1-6**) in the presence of DMAPP. (A) naringenin (**1**). (B) hesperitin (**2**), (C) eriodictyol (**3**), (D) liquiritigenin (**4**), (E) rac-pinocembrin (**5**), (F) dihydrogenistein (**6**).

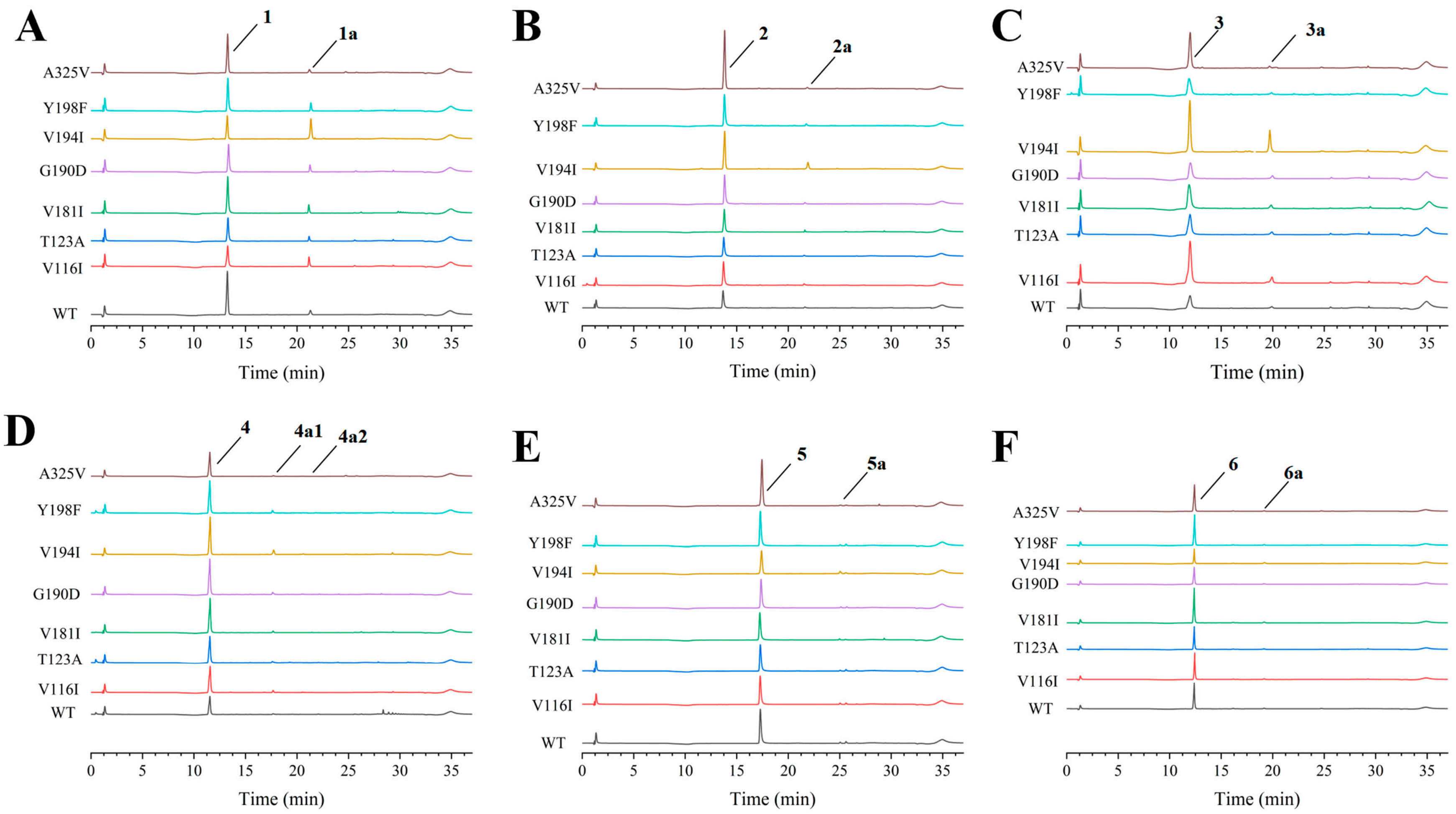

Figure 24. The activity of FgPT1 (wild type and 7 mutants) on substrates (**1-6**).

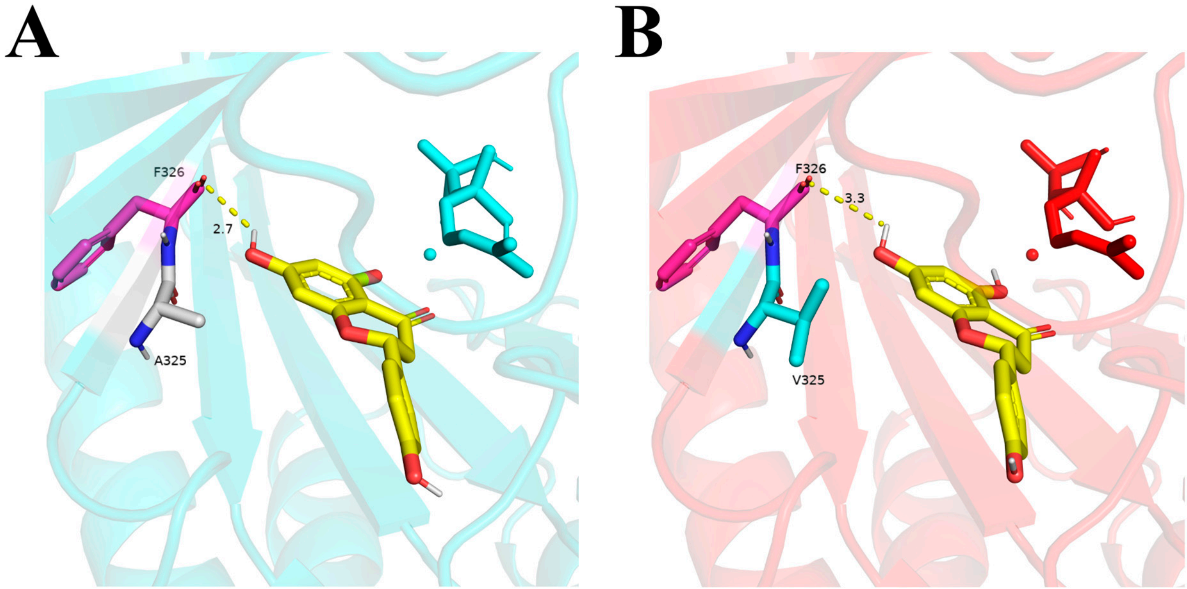

Figure S25. The docking model of FgPT1 (325 site). (A) naringenin, F326 and A325. (B) naringenin, F326 and V325.
